# Supplementary material for: Sperm competition risk drives rapid ejaculate adjustments mediated by seminal fluid
Source: eLife. 2017 Oct 31;6:e28811. doi: 10.7554/eLife.28811 (PMC5669631; doi:10.7554/eLife.28811)
Supplement: Supplementary file 1. [file elife-28811-supp1.docx]

**Statistical analysis and R code**

**Contents Page**

  Packages used 1
Models comparing ejaculate parameters in (D) and (S) males from stage 1 3
Models comparing ejaculate parameters in (D) and (S) males from stage 2 11
Models comparing changes in ejaculate parameters from stage 1 to 2 20
Seminal Fluid effect on sperm velocity 47
In-vitro fertilisation trials 55
Reference (packages) 66

**R^[1]^ (RRID:SCR_001905)** **Packages used:**

**library**(ggplot2)^[2]^ *#graphing* **(RRID:SCR_014601)**

**library**(lattice) ^[3]^ *#graphing* **(RRID:SCR_015662)**

**library**(lme4) ^[4]^ *#Linear mixed effects models* **(RRID:SCR_015654)**

**library**(nlme) ^[5]^ *#Linear mixed effects models with specified variance structures* **(RRID:SCR_015655)**

**library**(lmerTest) ^[6]^ *#P value approximations for lmer* **(RRID:SCR_015656)**

**library**(RVAideMemoire) ^[7]^ *#check for overdispersion in glmer* **(RRID:SCR_015657)**

**library**(LMERConvenienceFunctions) ^[8]^ *#partial regression plots for mixed effects models* **(RRID:SCR_015658)**

**library(**Deducer**)** ^[9]^ *#Fishers exact and G tests* **(RRID:SCR_015659)**

**Models comparing in ejaculate parameters (VAP or sperm count) in males of Dominant (D) and Subdominant (S) social status from stage 1 of social status manipulation:**

Linear mixed effects model with sperm velocity (VAP) as the response variable, social status and year as fixed predictors and male identity and week as random effects. Year was used as a fixed effect because it has fewer than 5 levels and is therefore unreliable as a random predictor^[10]^.

VAPDIFFSSA<-**read.table**(file="DIFF_VAP_SS_STAGE1.csv",header=T,row.names=NULL,sep=",")*#load Data sheet*

model1<-**lmer**(VAP ~ SOCIAL.STATUS + **as.factor**(YEAR) + (1|WEEK)+ (1|MALE.ID), data=VAPDIFFSSA)
**summary**(model1)

## Linear mixed model fit by REML t-tests use Satterthwaite approximations
##   to degrees of freedom [lmerMod]
## Formula:
## VAP ~ SOCIAL.STATUS + as.factor(YEAR) + (1 | WEEK) + (1 | MALE.ID)
##    Data: VAPDIFFSSA
##
## REML criterion at convergence: 699.3
##
## Scaled residuals:
##     Min      1Q  Median      3Q     Max
## -1.8588 -0.4541  0.0549  0.4637  1.9119
##
## Random effects:
##  Groups   Name        Variance Std.Dev.
##  MALE.ID  (Intercept) 712.46   26.692  
##  WEEK     (Intercept)   8.59    2.931  
##  Residual              40.90    6.395  
## Number of obs: 87, groups:  MALE.ID, 44; WEEK, 5
##
## Fixed effects:
##                          Estimate Std. Error      df t value Pr(>|t|)    
## (Intercept)               152.860      8.970  21.400  17.041 6.24e-14 ***
## SOCIAL.STATUSsubdominant    7.370      8.167  36.670   0.902    0.373    
## as.factor(YEAR)2014        11.824      9.942  39.960   1.189    0.241    
## as.factor(YEAR)2015        -1.832     11.130  38.810  -0.165    0.870    
## ---
## Signif. codes:  0 '***' 0.001 '**' 0.01 '*' 0.05 '.' 0.1 ' ' 1
##
## Correlation of Fixed Effects:
##              (Intr) SOCIAL a.(YEAR)2014
## SOCIAL.STAT  -0.455                    
## a.(YEAR)2014 -0.697  0.001             
## a.(YEAR)2015 -0.620  0.000  0.560

**rand**(model1)*#Test for significance of random predictor male ID*

## Analysis of Random effects Table:
##           Chi.sq Chi.DF p.value    
## WEEK      0.0168      1     0.9    
## MALE.ID 102.0088      1  <2e-16 ***
## ---
## Signif. codes:  0 '***' 0.001 '**' 0.01 '*' 0.05 '.' 0.1 ' ' 1

**confint**(model1, level=0.95, method="Wald",oldNames=F) *#generate 95%CI using Wald method*

##                               2.5 %    97.5 %
## sd_(Intercept)|MALE.ID           NA        NA
## sd_(Intercept)|WEEK              NA        NA
## sigma                            NA        NA
## (Intercept)              135.279207 170.44167
## SOCIAL.STATUSsubdominant  -8.636458  23.37664
## as.factor(YEAR)2014       -7.661824  31.31072
## as.factor(YEAR)2015      -23.646302  19.98171


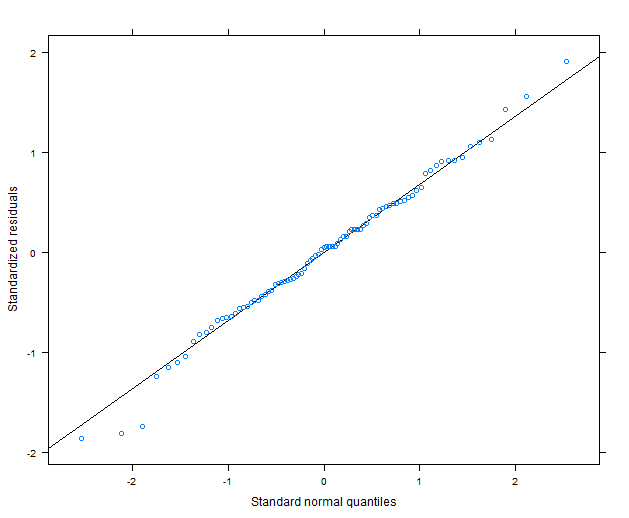
**qqmath**(model1) *#check normality assumption*

**shapiro.test**(**resid**(model1))
##  Shapiro-Wilk normality test
## data:  resid(model1)
## W = 0.99043, p-value = 0.7805

**plot**(model1, results="hide", fig.show='hide') *#plot residuals vs fitted values to check for unequal variance*


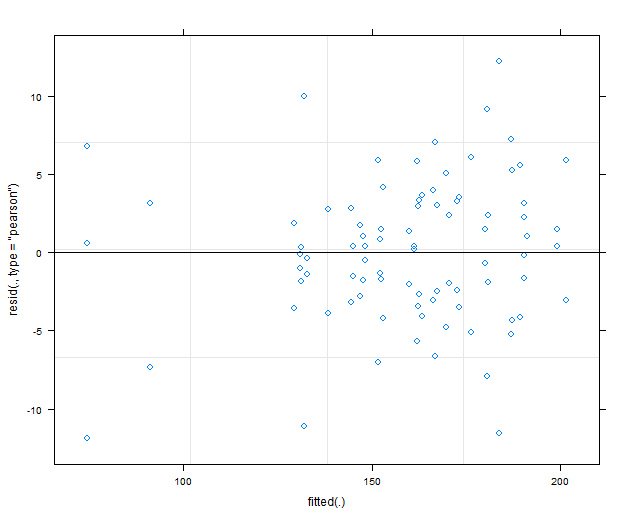


Variance structure may be violating assumptions

**plot**(VAPDIFFSSA$SOCIAL.STATUS, **resid**(model1), xlab="Status",ylab="Residuals")


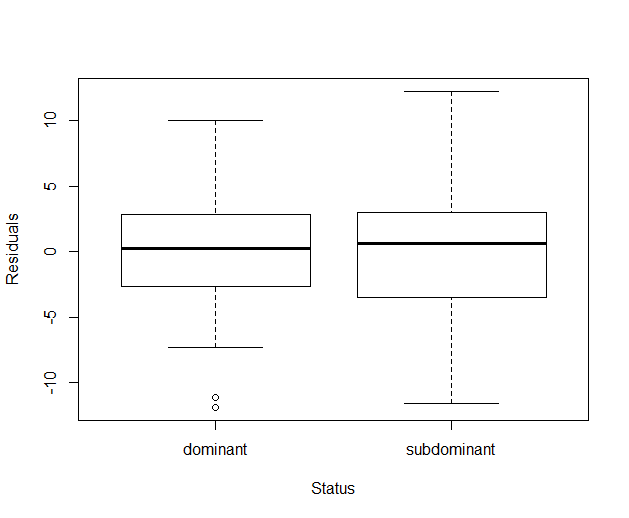


**Variance looks different for each social status group so will use lme() (nlme package) to implement different variances per level of status:**

vf1<-**varIdent**(form= ~ 1|SOCIAL.STATUS)
M2<-**lme**(VAP ~ SOCIAL.STATUS + **as.factor**(YEAR), data =VAPDIFFSSA,random = ~ 1|MALE.ID/WEEK, weights = vf1)
**summary**(M2)

## Linear mixed-effects model fit by REML
##  Data: VAPDIFFSSA
##        AIC      BIC    logLik
##   714.8842 734.2349 -349.4421
##
## Random effects:
##  Formula: ~1 | MALE.ID
##         (Intercept)
## StdDev:    19.00036
##
##  Formula: ~1 | WEEK %in% MALE.ID
##         (Intercept) Residual
## StdDev:    19.00036 6.838932
##
## Variance function:
##  Structure: Different standard deviations per stratum
##  Formula: ~1 | SOCIAL.STATUS
##  Parameter estimates:
## subdominant    dominant
##   1.0000000   0.8711948
## Fixed effects: VAP ~ SOCIAL.STATUS + as.factor(YEAR)
##                              Value Std.Error DF   t-value p-value
## (Intercept)              152.79580  8.864948 43 17.235950  0.0000
## SOCIAL.STATUSsubdominant   7.35512  8.221338 40  0.894638  0.3763
## as.factor(YEAR)2014       11.87273  9.954018 40  1.192758  0.2400
## as.factor(YEAR)2015       -2.14624 11.123055 40 -0.192954  0.8480
##  Correlation:
##                          (Intr) SOCIAL a.(YEAR)2014
## SOCIAL.STATUSsubdominant -0.462                    
## as.factor(YEAR)2014      -0.701  0.001             
## as.factor(YEAR)2015      -0.627  0.000  0.559      
##
## Standardized Within-Group Residuals:
##         Min          Q1         Med          Q3         Max
## -1.96121718 -0.48028457  0.03512917  0.46172327  1.79543625
##
## Number of Observations: 87
## Number of Groups:
##           MALE.ID WEEK %in% MALE.ID
##                44                44

**Estimates for fixed effects are the same as for lmer() (lme4) so will report stats from simpler model.**

Generalised Linear mixed effects model (Poisson) with sperm count as the response variable, social status and year as fixed predictors week as a random effect.

CountA<-**read.table**(file="DIFF_COUNT_SS_STAGE1.csv",header=T,row.names=NULL,sep=",")*#load data frame*

mod1<-**glmer**(COUNTRAW ~ **as.factor**(YEAR)+ SOCIAL.STATUS  + (1|WEEK),  data=CountA, family="poisson")

## Residual deviance: 1430.696 on 37 degrees of freedom (ratio: 38.667)

dispersion <- 1:**length**(CountA$YEAR) *#dispersion parameter for overdispersed model*

mod1.a<-**glmer**(COUNTRAW ~ **as.factor**(YEAR)+ SOCIAL.STATUS  + (1|WEEK) + (1|dispersion),  data=CountA,family="poisson") *#model was overdispersed so added dispersion parameter to correct for overdispersion.*
**summary**(mod1.a)

## Generalized linear mixed model fit by maximum likelihood (Laplace
##   Approximation) [glmerMod]
##  Family: poisson  ( log )
## Formula: COUNTRAW ~ as.factor(YEAR) + SOCIAL.STATUS + (1 | WEEK) + ## (1| dispersion)  
##     
##    Data: CountA
##
##      AIC      BIC   logLik deviance df.resid
##    532.0    542.4   -260.0    520.0       36
##
## Scaled residuals:
##      Min       1Q   Median       3Q      Max
## -0.45622 -0.15255  0.01699  0.10289  0.24218
##
## Random effects:
##  Groups     Name        Variance  Std.Dev.
##  dispersion (Intercept) 9.534e-02 3.088e-01
##  WEEK       (Intercept) 1.036e-10 1.018e-05
## Number of obs: 42, groups:  dispersion, 42; WEEK, 5
##
## Fixed effects:
##                          Estimate Std. Error z value Pr(>|z|)    
## (Intercept)               6.01889    0.10258   58.68  < 2e-16 ***
## as.factor(YEAR)2014      -0.43638    0.11691   -3.73 0.000189 ***
## as.factor(YEAR)2015       0.01945    0.12757    0.15 0.878805    
## SOCIAL.STATUSSubdominant  0.19858    0.09694    2.05 0.040519 *  
## ---
## Signif. codes:  0 '***' 0.001 '**' 0.01 '*' 0.05 '.' 0.1 ' ' 1
##
## Correlation of Fixed Effects:
##              (Intr) a.(YEAR)2014 a.(YEAR)2015
## a.(YEAR)2014 -0.701                          
## a.(YEAR)2015 -0.623  0.546                   
## SOCIAL.STAT  -0.476  0.047        0.002

**overdisp.glmer**(mod1.a)*# Now underdispersed*

## Residual deviance: 1.297 on 36 degrees of freedom (ratio: 0.036)

**confint**(mod1.a, level=0.95, method="Wald",oldNames=F) *#generate 95%CI using Wald method*

##                                  2.5 %     97.5 %
## sd_(Intercept)|dispersion           NA         NA
## sd_(Intercept)|WEEK                 NA         NA
## (Intercept)                5.817846528  6.2199398
## as.factor(YEAR)2014       -0.665519196 -0.2072460
## as.factor(YEAR)2015       -0.230583968  0.2694893
## SOCIAL.STATUSSubdominant   0.008575323  0.3885871

*#Test significance of random effects*
mod1.a2<-**glmer**(COUNTRAW ~ **as.factor**(YEAR)+ SOCIAL.STATUS + (1|dispersion),  data=CountA,family="poisson")
**anova**(mod1.a,mod1.a2)

## Data: CountA
## Models:
## mod1.a2: COUNTRAW ~ as.factor(YEAR) + SOCIAL.STATUS + (1 | dispersion)
## mod1.a: COUNTRAW ~ as.factor(YEAR) + SOCIAL.STATUS + (1 | WEEK) + (1 |
## mod1.a:     dispersion)
##         Df AIC    BIC logLik deviance Chisq Chi Df Pr(>Chisq)
## mod1.a2  5 530 538.69   -260      520                        
## mod1.a   6 532 542.43   -260      520     0      1     0.9999

**Models comparing ejaculate parameters (VAP or sperm count) in males of Dominant (D) and Subdominant (S) social status from stage 2 of social status manipulation:**

Linear mixed effects model with sperm velocity (VAP) as the response variable, social status and year as fixed predictors and male identity and week as random effects.

VAPDIFFSSB<-**read.table**(file=" DIFF_VAP_SS_STAGE2.csv",header=T,row.names=NULL,sep=",")*#Load data frame*

**Start with social status as factor with 2 levels, S (SS and DS pooled) and D (DD and SD pooled).**

model2<-**lmer**(VAP ~ **as.factor**(YEAR) + SOCIAL.STATUS + (1|WEEK)+ (1|MALE.ID), data=VAPDIFFSSB) *#D and S males pooled*
**summary**(model2)

## Linear mixed model fit by REML t-tests use Satterthwaite approximations
##   to degrees of freedom [lmerMod]
## Formula:
## VAP ~ as.factor(YEAR) + SOCIAL.STATUS + (1 | WEEK) + (1 | MALE.ID)
##    Data: VAPDIFFSSB
##
## REML criterion at convergence: 581.3
##
## Scaled residuals:
##      Min       1Q   Median       3Q      Max
## -1.93909 -0.48952 -0.00969  0.58019  1.92393
##
## Random effects:
##  Groups   Name        Variance Std.Dev.
##  MALE.ID  (Intercept) 503.62   22.441  
##  WEEK     (Intercept)  75.83    8.708  
##  Residual              32.73    5.721  
## Number of obs: 75, groups:  MALE.ID, 38; WEEK, 5
##
## Fixed effects:
##                          Estimate Std. Error      df t value Pr(>|t|)    
## (Intercept)               127.147      9.373  18.030  13.566 6.68e-11 ***
## as.factor(YEAR)2014        34.192      9.633  34.010   3.550  0.00115 **
## as.factor(YEAR)2015        29.836     10.421  33.640   2.863  0.00717 **
## SOCIAL.STATUSsubdominant   19.653      7.401  30.870   2.655  0.01242 *  
## ---
## Signif. codes:  0 '***' 0.001 '**' 0.01 '*' 0.05 '.' 0.1 ' ' 1
##
## Correlation of Fixed Effects:
##              (Intr) a.(YEAR)2014 a.(YEAR)2015
## a.(YEAR)2014 -0.656                          
## a.(YEAR)2015 -0.606  0.593                   
## SOCIAL.STAT  -0.395  0.001        0.000

**confint**(model2, level=0.95, method="Wald",oldNames=F) *#generate 95%CI using Wald method*

##                               2.5 %    97.5 %
## sd_(Intercept)|MALE.ID           NA        NA
## sd_(Intercept)|WEEK              NA        NA
## sigma                            NA        NA
## (Intercept)              108.776923 145.51751
## as.factor(YEAR)2014       15.312380  53.07158
## as.factor(YEAR)2015        9.410564  50.26056
## SOCIAL.STATUSsubdominant   5.147170  34.15966

**qqmath**(model2) *#check normality assumption*
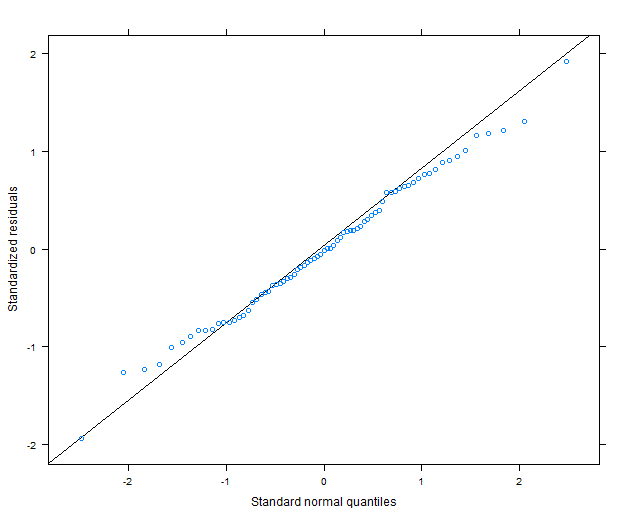


**shapiro.test**(**resid**(model2))
##  Shapiro-Wilk normality test
## data:  resid(model2)
## W = 0.99423, p-value = 0.9838

**plot**(model2, results="hide", fig.show='hide') *#plot residuals vs fitted values to check for unequal variance*


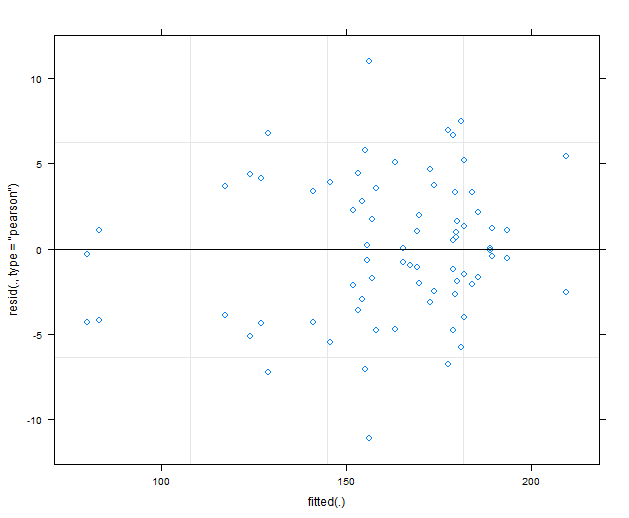


*#Test significance of random effects*
**rand**(model2)

## Analysis of Random effects Table:
##         Chi.sq Chi.DF p.value    
## WEEK      1.49      1     0.2    
## MALE.ID  76.91      1  <2e-16 ***
## ---
## Signif. codes:  0 '***' 0.001 '**' 0.01 '*' 0.05 '.' 0.1 ' ' 1

**Now use factor SSGROUP with four levels (DD,DS,SD,SS).**

This model was run 3 times using the relevel code below^***^ to change the social status group that each other group is compared to, first using DD, then DS and then SD. The results are collated below.

e.g.***VAPDIFFSSB$SS.GROUP<-**relevel**(VAPDIFFSSB$SS.GROUP,"DD")*** # this model compares group DD to each other group.

model2.1<-**lmer**(VAP ~ **as.factor**(YEAR) + SS.GROUP + (1|WEEK)+ (1|MALE.ID), data=VAPDIFFSSB) *#four social status groups DD, SD, DS, SS*
**summary**(model2)

Linear mixed model fit by REML t-tests use Satterthwaite approximations to degrees of freedom [lmerMod]

Formula: VAP ~ as.factor(YEAR) + SS.GROUP + (1 | WEEK) + (1 | MALE.ID)

  Data: VAPDIFFSSB

REML criterion at convergence: 567.7

Scaled residuals:

    Min       1Q   Median       3Q      Max

-1.92015 -0.50118  0.00319  0.59748  1.94278

Random effects:

Groups   Name        Variance Std.Dev.

MALE.ID  (Intercept) 535.73   23.146

WEEK     (Intercept)  66.02    8.125

Residual              32.73    5.721

Number of obs: 75, groups:  MALE.ID, 38; WEEK, 5

Fixed effects:

                   Estimate Std. Error      df t value Pr(>|t|)

(Intercept)          131.232     11.227  24.030  11.689 2.11e-11 ***

as.factor(YEAR)2014   32.621     10.076  31.970   3.238  0.00281 **

as.factor(YEAR)2015   29.003     10.708  31.530   2.708  0.01083 *

SS.GROUP DD - DS     17.895     10.516  29.070   1.702  0.09947 .

SS.GROUP DD - SD     -6.513     10.969  29.780  -0.594  0.55717

SS.GROUP DD - SS     14.982     10.969  29.780   1.366  0.18222

SS.GROUP DS - SD    -24.408     10.974  29.830  -2.224  0.03386 *

SS.GROUP DS - SS     -2.914     10.974  29.830  -0.265  0.79246

SS.GROUP SD - SS     21.494     11.076  28.990   1.941  0.06209 .

---

Signif. codes:  0 ‘***’ 0.001 ‘**’ 0.01 ‘*’ 0.05 ‘.’ 0.1 ‘ ’ 1

No significant results after applying p value correction (alpha = 0.016)

Correlation of Fixed Effects:

            (Intr) a.(YEAR)2014 a.(YEAR)2015 SS.GROUP D SS.GROUP SD

a.(YEAR)2014 -0.634

a.(YEAR)2015 -0.555  0.596

SS.GROUP DS  -0.468  0.001        0.000

SS.GROUP SD  -0.534  0.144        0.067        0.479

SS.GROUP SS  -0.534  0.144        0.067        0.479      0.490

**confint**(model2.1, level=0.95, method="Wald",oldNames=F) *#generate 95%CI using Wald method*

                           2.5 %    97.5 %

sd_(Intercept)|MALE.ID         NA        NA

sd_(Intercept)|WEEK            NA        NA

sigma                          NA        NA

(Intercept)            109.227477 153.23556

as.factor(YEAR)2014     12.873665  52.36927

as.factor(YEAR)2015      8.014878  49.99089

SS.GROUP DD – DS        -2.715183  38.50554

SS.GROUP DD - SD       -28.011627  14.98605

SS.GROUP DD - SS        -6.517183  36.48049

SS.GROUP DS - SD       -45.916823  -2.899110

SS.GROUP DS - SS       -24.422378  18.595334

SS.GROUP SD - SS        -0.215076  43.20396

**qqmath**(model2) *#check normality assumption*


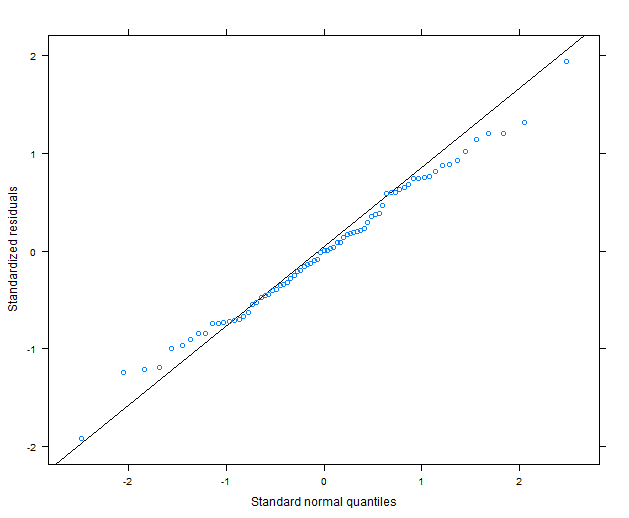


**shapiro.test**(**resid**(model2.1))

Shapiro-Wilk normality test

data:  resid(model2)

W = 0.9931, p-value = 0.961

**plot**(model2.1, results="hide", fig.show='hide') *#plot residuals vs fitted values to check for unequal variance*


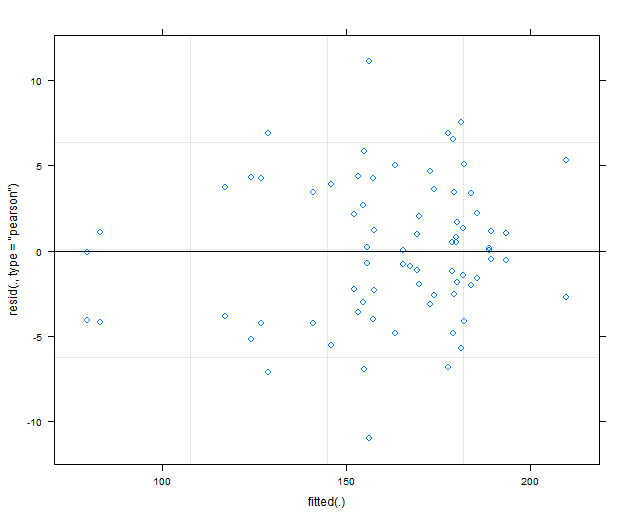


Generalised linear mixed effects model with sperm count as the response variable, social status and year as fixed predictors and week as a random effect. A poisson error distribution is used for count data

CountB<-**read.table**(file=" DIFF_COUNT_SS_STAGE2.csv",header=T,row.names=NULL,sep=",")*#load data frame*

**Start with social status as factor with 2 levels, S (SS and DS pooled) and D (DD and SD pooled).**

mod2<-**glmer**(COUNTRAW ~  **as.factor**(YEAR) + SOCIAL.STATUS  + (1|WEEK),  data=CountB, family="poisson")

**overdisp.glmer**(mod2)*# check for over/under-dispersion*

## Residual deviance: 2087.387 on 34 degrees of freedom (ratio: 61.394)

dispersion2<-1:**length**(CountB$YEAR)*# disperion parameter for overdispersed model*

mod2.a<-**glmer**(COUNTRAW ~  **as.factor**(YEAR) + SOCIAL.STATUS  + (1|WEEK) + (1|dispersion2),  data=CountB, family="poisson") *#model overdispersed so added dispersion parameter*
**summary**(mod2.a)

## Generalized linear mixed model fit by maximum likelihood (Laplace
##   Approximation) [glmerMod]
##  Family: poisson  ( log )
## Formula: COUNTRAW ~ as.factor(YEAR) + SOCIAL.STATUS + (1 | WEEK) + ## (1 | dispersion2)     
##    Data: CountB
##
##      AIC      BIC   logLik deviance df.resid
##    501.4    511.4   -244.7    489.4       33
##
## Scaled residuals:
##      Min       1Q   Median       3Q      Max
## -0.30444 -0.13054 -0.01825  0.09442  0.20362
##
## Random effects:
##  Groups      Name        Variance Std.Dev.
##  dispersion2 (Intercept) 0.1365   0.3694  
##  WEEK        (Intercept) 0.0000   0.0000  
## Number of obs: 39, groups:  dispersion2, 39; WEEK, 5
##
## Fixed effects:
##                          Estimate Std. Error z value Pr(>|z|)    
## (Intercept)                5.9688     0.1250   47.77   <2e-16 ***
## as.factor(YEAR)2014       -0.3301     0.1463   -2.26   0.0241 *  
## as.factor(YEAR)2015       -0.2475     0.1559   -1.59   0.1125    
## SOCIAL.STATUSSubdominant   0.1754     0.1198    1.46   0.1431    
## ---
## Signif. codes:  0 '***' 0.001 '**' 0.01 '*' 0.05 '.' 0.1 ' ' 1
##
## Correlation of Fixed Effects:
##              (Intr) a.(YEAR)2014 a.(YEAR)2015
## a.(YEAR)2014 -0.675                          
## a.(YEAR)2015 -0.633  0.555                   
## SOCIAL.STAT  -0.436 -0.037       -0.036

**overdisp.glmer**(mod2.a)*# check for over/under-dispersion*

## Residual deviance: 0.77 on 33 degrees of freedom (ratio: 0.023)

**confint**(mod2.a, level=0.95, method="Wald",oldNames=F) *#generate 95%CI using Wald method*

##                                  2.5 %      97.5 %
## sd_(Intercept)|dispersion2          NA          NA
## sd_(Intercept)|WEEK                 NA          NA
## (Intercept)                 5.72387849  6.21371400
## as.factor(YEAR)2014        -0.61685909 -0.04326561
## as.factor(YEAR)2015        -0.55304392  0.05810625
## SOCIAL.STATUSSubdominant   -0.05937055  0.41014065

**Now use factor SSGROUP with four levels (DD,DS,SD,SS).**

This model was run 3 times using the relevel code below^***^ to change the social status group that each other group is compared to, first using DD, then DS and then SD. The results are collated below.

***CountB$SSGROUP <-**relevel**(CountB$SSGROUP,"DD")***

mod2.b<-**glmer**(COUNTRAW ~  **as.factor**(YEAR) + SSGROUP  + (1|WEEK) + (1|dispersion2),  data=CountB, family="poisson") *#model overdispersed so added dispersion parameter*
**summary**(mod2.b)

## Generalized linear mixed model fit by maximum likelihood (Laplace
##   Approximation) [glmerMod]
##  Family: poisson  ( log )
## Formula:
## COUNTRAW ~ as.factor(YEAR) + SSGROUP + (1 | WEEK) + (1 | dispersion2)
##    Data: CountB
##
##      AIC      BIC   logLik deviance df.resid
##    498.9    512.2   -241.5    482.9       31
##
## Scaled residuals:
##      Min       1Q   Median       3Q      Max
## -0.34875 -0.15248 -0.00253  0.08914  0.24087
##
## Random effects:
##  Groups      Name        Variance Std.Dev.
##  dispersion2 (Intercept) 0.1151   0.3392  
##  WEEK        (Intercept) 0.0000   0.0000  
## Number of obs: 39, groups:  dispersion2, 39; WEEK, 5
##
## Fixed effects:
##                     Estimate Std. Error z value Pr(>|z|)    
## (Intercept)           5.8232     0.1373   42.42  < 2e-16 ***
## as.factor(YEAR)2014  -0.2773     0.1361   -2.04  0.04159 *  
## as.factor(YEAR)2015  -0.2318     0.1436   -1.61  0.10651    
## SSGROUP DD - DS      0.1388     0.1507    0.92  0.35709    
## SSGROUP DD - SD    0.2665     0.1552    1.72  0.08606 .  
## SSGROUP DD - SS      0.4659     0.1550    3.01  0.00265 **
## SSGROUP DS - SD 0.1277 0.1593 0.80 0.4227
## SSGROUP DS – SS 0.3271 0.1590 2.06 0.0397 *
## SSGROUP SD – SS -0.1994 0.1617 -1.23 0.21745
## ---
## Signif. codes:  0 '***' 0.001 '**' 0.01 '*' 0.05 '.' 0.1 ' ' 1

**DD – SS significant** result after applying p value correction (alpha = 0.016)

## Correlation of Fixed Effects:
##              (Intr) a.(YEAR)2014 a.(YEAR)2015 SSGROUPDS SSGROUPSD
## a.(YEAR)2014 -0.610                                             
## a.(YEAR)2015 -0.535  0.555                                      
## SSGROUP DS   -0.483 -0.054       -0.051                         
## SSGROUP SD -0.543  0.078        0.004        0.458            
## SSGROUP SS -0.543  0.077        0.003        0.459       0.457

**overdisp.glmer**(mod2.b)*# check for over/under-dispersion*

## Residual deviance: 0.892 on 31 degrees of freedom (ratio: 0.029)

**confint**(mod2.b, level=0.95, method="Wald",oldNames=F) *#generate 95%CI using Wald method*

##                                  2.5 %      97.5 %
## sd_(Intercept)|dispersion2          NA          NA
## sd_(Intercept)|WEEK                 NA          NA
## (Intercept)                 5.55411444  6.09225136
## as.factor(YEAR)2014        -0.54411128 -0.01056046
## as.factor(YEAR)2015        -0.51331918  0.04967555
## SSGROUP DD - DS       -0.15659105  0.43417286
## SSGROUP DD - SD          -0.03778601  0.57075869
## SSGROUP DD - SS           0.16210528  0.76975328
## SSGROUP DS – SD -0.18448161 0.43988726
## SSGROUP DS – SS 0.01540979 0.63887362
## SSGROUP SD – SS -0.5163898 0.11750514

**Models to compare changes in ejaculate parameters (VAP or sperm count) in males from each social group from stage 1 to 2:**

Model that compares mean VAP for males that are D (stage 1) that become DS (stage 2).

CHANGEDTODSVAP<-**read.table**(file=" CHANGE_VAP_D_TO_DD.csv",header=T,row.names=NULL,sep=",")*#load data frame*

model4<-**lmer**(VAP ~ **as.factor**(YEAR) + SOCIAL.STATUS + (1|WEEK) + (1|MALE.ID) ,data=CHANGEDTODSVAP)
**summary**(model4)

## Linear mixed model fit by REML t-tests use Satterthwaite approximations
##   to degrees of freedom [lmerMod]
## Formula:
## VAP ~ as.factor(YEAR) + SOCIAL.STATUS + (1 | WEEK) + (1 | MALE.ID)
##    Data: CHANGEDTODSVAP
##
## REML criterion at convergence: 303
##
## Scaled residuals:
##      Min       1Q   Median       3Q      Max
## -2.14236 -0.41628 -0.06181  0.69382  1.67821
##
## Random effects:
##  Groups   Name        Variance Std.Dev.
##  MALE.ID  (Intercept)   2.851   1.689  
##  WEEK     (Intercept) 345.651  18.592  
##  Residual             333.371  18.258  
## Number of obs: 37, groups:  MALE.ID, 10; WEEK, 5
##
## Fixed effects:
##                     Estimate Std. Error      df t value Pr(>|t|)    
## (Intercept)          163.920     11.667   6.878  14.050 2.56e-06 ***
## as.factor(YEAR)2014  -13.500      8.970   3.299  -1.505   0.2213    
## as.factor(YEAR)2015  -10.565     10.099   3.283  -1.046   0.3663    
## SOCIAL.STATUSDS       17.242      6.050  26.292   2.850   0.0084 **
## ---
## Signif. codes:  0 '***' 0.001 '**' 0.01 '*' 0.05 '.' 0.1 ' ' 1

**remains significant following P-value correction (alpha = 0.0125)
##
## Correlation of Fixed Effects:
##              (Intr) a.(YEAR)2014 a.(YEAR)2015
## a.(YEAR)2014 -0.569                          
## a.(YEAR)2015 -0.515  0.685                   
## SOCIAL.STAT  -0.228 -0.019       -0.062

**confint**(model4, level=0.95, method="Wald",oldNames=F) *#generate 95%CI using Wald method*

                                2.5 %     97.5 %
## sd_(Intercept)|MALE.ID         NA         NA
## sd_(Intercept)|WEEK            NA         NA
## sigma                          NA         NA
## (Intercept)            141.053996 186.786069
## as.factor(YEAR)2014    -31.081606   4.081081
## as.factor(YEAR)2015    -30.358576   9.228218
## SOCIAL.STATUSDS          5.383677  29.100929

**qqmath**(model4) *#check normality assumption*


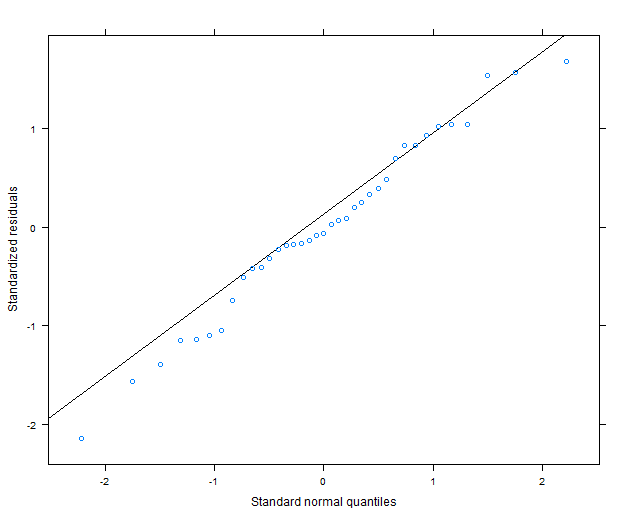


**shapiro.test**(**resid**(model4))
##  Shapiro-Wilk normality test
## data:  resid(model4)
## W = 0.98145, p-value = 0.7808

**plot**(model4, results="hide", fig.show='hide') *#plot residuals vs fitted values to check for unequal variance*


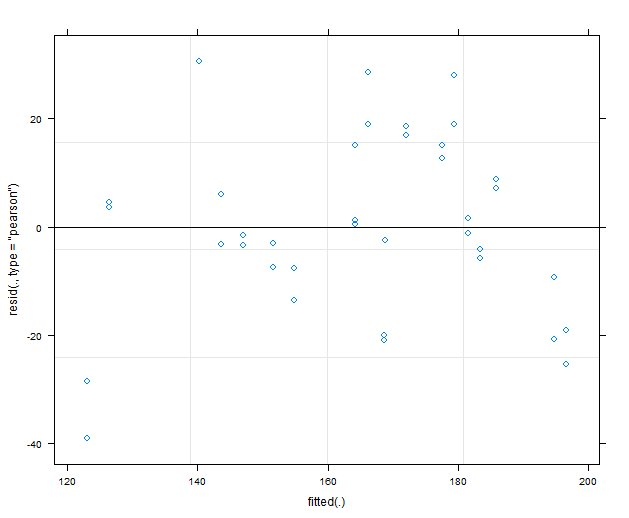


*#Test for significance of random predictor male ID*
model4.1<-**lmer**(VAP ~ **as.factor**(YEAR) + SOCIAL.STATUS + (1|WEEK) ,data=CHANGEDTODSVAP)
**anova**(model4,model4.1)

## refitting model(s) with ML (instead of REML)

## Data: CHANGEDTODSVAP
## Models:
## ..1: VAP ~ as.factor(YEAR) + SOCIAL.STATUS + (1 | WEEK)
## object: VAP ~ as.factor(YEAR) + SOCIAL.STATUS + (1 | WEEK) + (1 | MALE.ID)
##        Df    AIC    BIC  logLik deviance Chisq Chi Df Pr(>Chisq)
## ..1     6 338.36 348.02 -163.18   326.36                        
## object  7 340.36 351.63 -163.18   326.36     0      1          1

Model that compares mean VAP for males that are D (stage 1) that become DD (stage 2).

CHANGEDTODDVAP<-**read.table**(file=" CHANGE_VAP_D_TO_DD.csv",header=T,row.names=NULL,sep=",")*#load data frame*

model5<-**lmer**(VAP ~ **as.factor**(YEAR) + SOCIAL.STATUS  + (1|WEEK) + (1|MALE.ID) ,data=CHANGEDTODDVAP)
**summary**(model5)

## Linear mixed model fit by REML t-tests use Satterthwaite approximations
##   to degrees of freedom [lmerMod]
## Formula:
## VAP ~ as.factor(YEAR) + SOCIAL.STATUS + (1 | WEEK) + (1 | MALE.ID)
##    Data: CHANGEDTODDVAP
##
## REML criterion at convergence: 320.1
##
## Scaled residuals:
##     Min      1Q  Median      3Q     Max
## -1.4747 -0.5969 -0.0803  0.5212  2.0354
##
## Random effects:
##  Groups   Name        Variance Std.Dev.
##  MALE.ID  (Intercept)  60.49    7.777  
##  WEEK     (Intercept)   0.00    0.000  
##  Residual             499.21   22.343  
## Number of obs: 38, groups:  MALE.ID, 10; WEEK, 5
##
## Fixed effects:
##                     Estimate Std. Error       df t value Pr(>|t|)    
## (Intercept)         109.0623    10.2910   8.2350  10.598 4.41e-06 ***
## as.factor(YEAR)2014  60.0995    11.5247   6.5910   5.215  0.00149 **
## as.factor(YEAR)2015  60.4750    12.4261   6.3210   4.867  0.00243 **
## SOCIAL.STATUSDD       0.1255     7.2826  27.3450   0.017  0.98638    
## ---
## Signif. codes:  0 '***' 0.001 '**' 0.01 '*' 0.05 '.' 0.1 ' ' 1
##
## Correlation of Fixed Effects:
##              (Intr) a.(YEAR)2014 a.(YEAR)2015
## a.(YEAR)2014 -0.768                          
## a.(YEAR)2015 -0.724  0.647                   
## SOCIAL.STAT  -0.354 -0.037        0.000

**confint**(model5, level=0.95, method="Wald",oldNames=F) *#generate 95%CI using Wald method*

##                            2.5 %    97.5 %
## sd_(Intercept)|MALE.ID        NA        NA
## sd_(Intercept)|WEEK           NA        NA
## sigma                         NA        NA
## (Intercept)             88.89237 129.23217
## as.factor(YEAR)2014     37.51157  82.68750
## as.factor(YEAR)2015     36.12032  84.82968
## SOCIAL.STATUSDD        -14.14826  14.39919

**qqmath**(model5) *#check normality assumption*


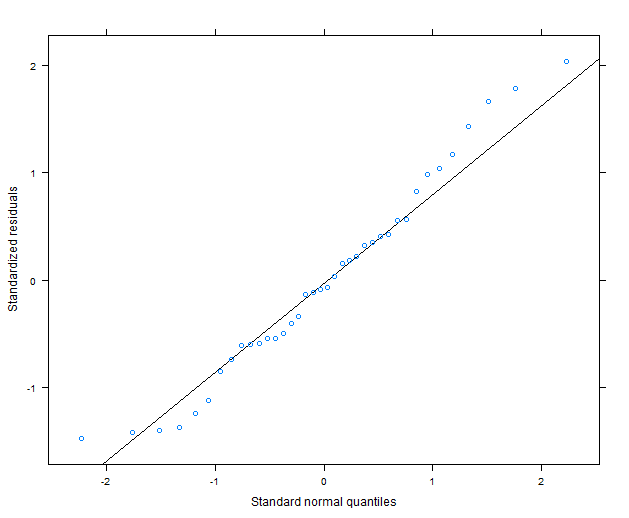


**shapiro.test**(**resid**(model5))
##  Shapiro-Wilk normality test
## data:  resid(model5)
## W = 0.96978, p-value = 0.3857

**plot**(model5, results="hide", fig.show='hide') *#plot residuals vs fitted values to check for unequal variance*


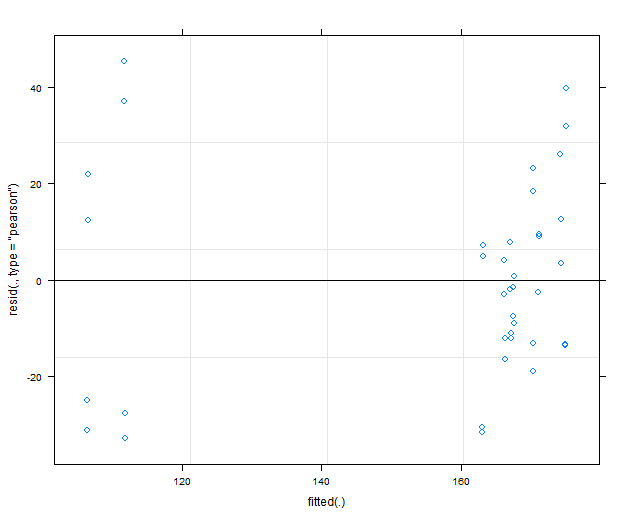


*#Test for significance of random predictor male ID*
model5.1<-**lmer**(VAP ~ **as.factor**(YEAR) + SOCIAL.STATUS  + (1|WEEK) ,data=CHANGEDTODDVAP)
**anova**(model5,model5.1)

## refitting model(s) with ML (instead of REML)

## Data: CHANGEDTODDVAP
## Models:
## ..1: VAP ~ as.factor(YEAR) + SOCIAL.STATUS + (1 | WEEK)
## object: VAP ~ as.factor(YEAR) + SOCIAL.STATUS + (1 | WEEK) + (1 | MALE.ID)
##        Df    AIC    BIC  logLik deviance  Chisq Chi Df Pr(>Chisq)
## ..1     6 355.10 364.92 -171.55   343.10                         
## object  7 357.09 368.56 -171.55   343.09 0.0043      1     0.9476

Model that compares mean VAP for males that are S (stage 1) that become SD (stage 2).

CHANGESTOSDVAP<-**read.table**(file=" CHANGE_VAP_S_TO_SD.csv",header=T,row.names=NULL,sep=",")*#load data frame*
model6<-**lmer**(VAP ~  **as.factor**(YEAR) +SOCIAL.STATUS+ (1|WEEK) + (1|MALE.ID) ,data=CHANGESTOSDVAP)
**summary**(model6)

## Linear mixed model fit by REML t-tests use Satterthwaite approximations
##   to degrees of freedom [lmerMod]
## Formula:
## VAP ~ as.factor(YEAR) + SOCIAL.STATUS + (1 | WEEK) + (1 | MALE.ID)
##    Data: CHANGESTOSDVAP
##
## REML criterion at convergence: 291.6
##
## Scaled residuals:
##      Min       1Q   Median       3Q      Max
## -1.80659 -0.66496  0.02879  0.67560  1.50712
##
## Random effects:
##  Groups   Name        Variance Std.Dev.
##  MALE.ID  (Intercept) 511.3    22.61   
##  WEEK     (Intercept)   0.0     0.00   
##  Residual             261.0    16.16   
## Number of obs: 36, groups:  MALE.ID, 9; WEEK, 5
##
## Fixed effects:
##                     Estimate Std. Error      df t value Pr(>|t|)    
## (Intercept)          139.553     14.122   6.459   9.882  3.9e-05 ***
## as.factor(YEAR)2014   30.300     19.605   6.000   1.546    0.173    
## as.factor(YEAR)2015   14.742     19.605   6.000   0.752    0.481    
## SOCIAL.STATUSSD       -8.972      5.385  26.000  -1.666    0.108    
## ---
## Signif. codes:  0 '***' 0.001 '**' 0.01 '*' 0.05 '.' 0.1 ' ' 1
##
## Correlation of Fixed Effects:
##              (Intr) a.(YEAR)2014 a.(YEAR)2015
## a.(YEAR)2014 -0.694                          
## a.(YEAR)2015 -0.694  0.500                   
## SOCIAL.STAT  -0.191  0.000        0.000

**confint**(model6, level=0.95, method="Wald",oldNames=F) *#generate 95%CI using Wald method*

##                             2.5 %     97.5 %
## sd_(Intercept)|MALE.ID         NA         NA
## sd_(Intercept)|WEEK            NA         NA
## sigma                          NA         NA
## (Intercept)            111.874737 167.230819
## as.factor(YEAR)2014     -8.124501  68.724501
## as.factor(YEAR)2015    -23.682835  53.166168
## SOCIAL.STATUSSD        -19.527376   1.582931

**qqmath**(model6) *#check normality assumption*


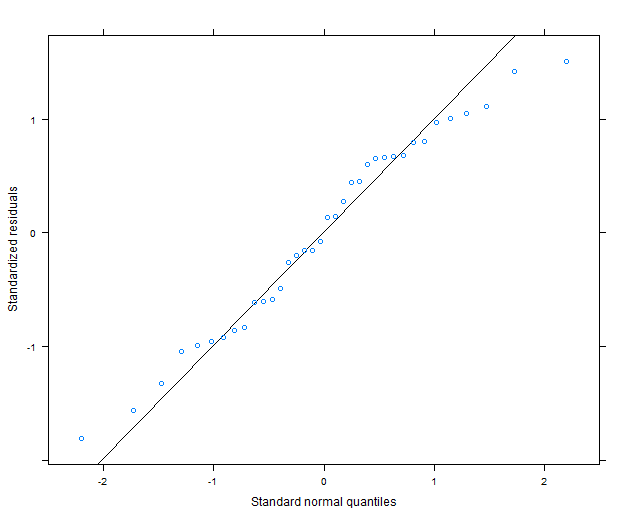


**shapiro.test**(**resid**(model6))

##  Shapiro-Wilk normality test
## data:  resid(model6)
## W = 0.96772, p-value = 0.3665

**plot**(model6, results="hide", fig.show='hide') *#plot residuals vs fitted values to check for unequal variance*


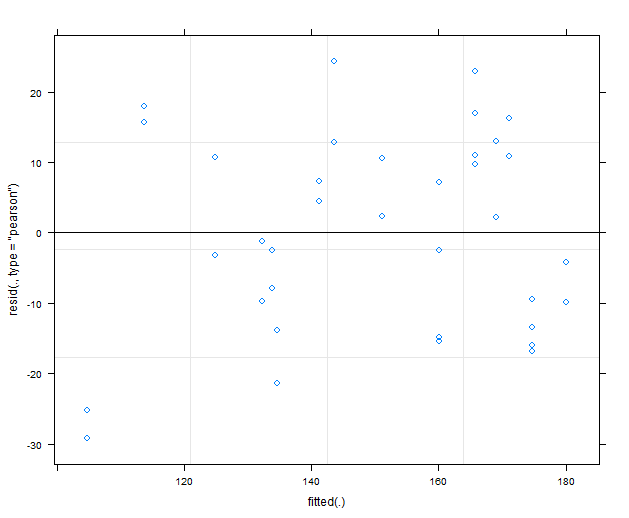


*#Test for significance of random predictor male ID*
model6.1<-**lmer**(VAP ~  **as.factor**(YEAR) +SOCIAL.STATUS+ (1|WEEK) ,data=CHANGESTOSDVAP)
**anova**(model6,model6.1)

## refitting model(s) with ML (instead of REML)

## Data: CHANGESTOSDVAP
## Models:
## ..1: VAP ~ as.factor(YEAR) + SOCIAL.STATUS + (1 | WEEK)
## object: VAP ~ as.factor(YEAR) + SOCIAL.STATUS + (1 | WEEK) + (1 | MALE.ID)
##        Df    AIC    BIC  logLik deviance  Chisq Chi Df Pr(>Chisq)    
## ..1     6 341.67 351.17 -164.84   329.67                             
## object  7 331.43 342.51 -158.72   317.43 12.242      1  0.0004672 ***
## ---
## Signif. codes:  0 '***' 0.001 '**' 0.01 '*' 0.05 '.' 0.1 ' ' 1

Model that compares mean VAP for males that are S (stage 1) that become SS (stage 2).

CHANGESTOSSVAP<-**read.table**(file=" CHANGE_VAP_S_TO_SS.csv",header=T,row.names=NULL,sep=",")*#load data frame*

model7<-**lmer**(VAP ~  **as.factor**(YEAR) +SOCIAL.STATUS+ (1|WEEK) + (1|MALE.ID) ,data=CHANGESTOSSVAP)
**summary**(model7)

## Linear mixed model fit by REML t-tests use Satterthwaite approximations
##   to degrees of freedom [lmerMod]
## Formula:
## VAP ~ as.factor(YEAR) + SOCIAL.STATUS + (1 | WEEK) + (1 | MALE.ID)
##    Data: CHANGESTOSSVAP
##
## REML criterion at convergence: 261.2
##
## Scaled residuals:
##     Min      1Q  Median      3Q     Max
## -1.2715 -0.7319 -0.1309  0.5791  2.0023
##
## Random effects:
##  Groups   Name        Variance Std.Dev.
##  MALE.ID  (Intercept) 115.7    10.76   
##  WEEK     (Intercept)   0.0     0.00   
##  Residual             206.0    14.35   
## Number of obs: 34, groups:  MALE.ID, 9; WEEK, 5
##
## Fixed effects:
##                     Estimate Std. Error      df t value Pr(>|t|)    
## (Intercept)          162.469      7.867   7.132  20.652 1.26e-07 ***
## as.factor(YEAR)2014   11.205     10.646   5.986   1.053    0.333    
## as.factor(YEAR)2015   -1.153     10.646   5.986  -0.108    0.917    
## SOCIAL.STATUSSS       -2.322      4.960  24.301  -0.468    0.644    
## ---
## Signif. codes:  0 '***' 0.001 '**' 0.01 '*' 0.05 '.' 0.1 ' ' 1
##
## Correlation of Fixed Effects:
##              (Intr) a.(YEAR)2014 a.(YEAR)2015
## a.(YEAR)2014 -0.658                          
## a.(YEAR)2015 -0.673  0.491                   
## SOCIAL.STAT  -0.315 -0.024        0.024

**confint**(model7, level=0.95, method="Wald",oldNames=F) *#generate 95%CI using Wald method*

##                             2.5 %     97.5 %
## sd_(Intercept)|MALE.ID         NA         NA
## sd_(Intercept)|WEEK            NA         NA
## sigma                          NA         NA
## (Intercept)            147.050721 177.888267
## as.factor(YEAR)2014     -9.659388  32.070375
## as.factor(YEAR)2015    -22.017870  19.711893
## SOCIAL.STATUSSS        -12.043283   7.398641

**qqmath**(model7) *#check normality assumption*


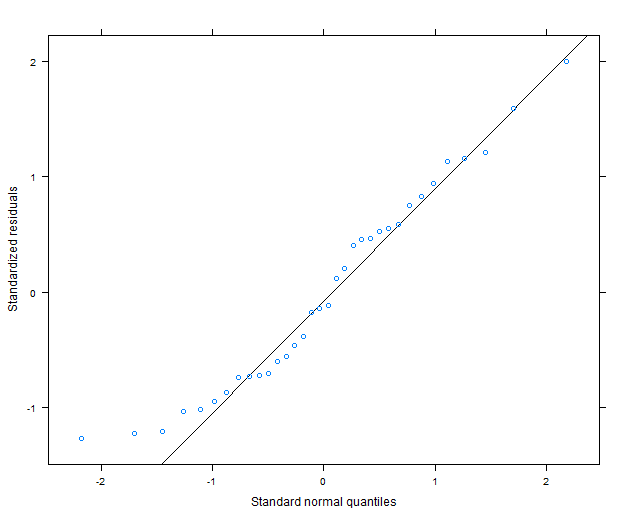


**shapiro.test**(**resid**(model7))
##  Shapiro-Wilk normality test
## data:  resid(model7)
## W = 0.95274, p-value = 0.1483

**plot**(model7, results="hide", fig.show='hide') *#plot residuals vs fitted values to check for unequal variance*


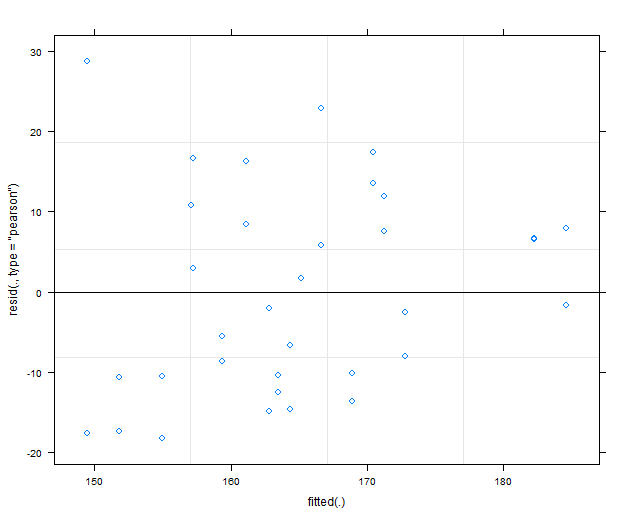


*#Test for significance of random predictor male ID*
model7.1<-**lmer**(VAP ~  **as.factor**(YEAR) +SOCIAL.STATUS+ (1|WEEK) ,data=CHANGESTOSSVAP)
**anova**(model7,model7.1)

## refitting model(s) with ML (instead of REML)

## Data: CHANGESTOSSVAP
## Models:
## ..1: VAP ~ as.factor(YEAR) + SOCIAL.STATUS + (1 | WEEK)
## object: VAP ~ as.factor(YEAR) + SOCIAL.STATUS + (1 | WEEK) + (1 | MALE.ID)
##        Df    AIC    BIC  logLik deviance Chisq Chi Df Pr(>Chisq)
## ..1     6 297.44 306.60 -142.72   285.44                        
## object  7 297.19 307.88 -141.60   283.19 2.252      1     0.1334

Models that compare sperm counts for males that are D (stage 1) that become DS (stage 2).

CHANGEDTODS<-**read.table**(file=" CHANGE_COUNT_D_TO_DS.csv",header=T,row.names=NULL,sep=",")*#load data frame*

mod4<-**glmer**(COUNTRAW ~ **as.factor**(YEAR) + SOCIAL.STATUS  + (1|WEEK) +  (1|MALE.ID) ,data=CHANGEDTODS,family="poisson")
**overdisp.glmer**(mod4)*# check for over/under-dispersion*

## Residual deviance: 267.048 on 14 degrees of freedom (ratio: 19.075)

dispersionDS<-1:**length**(CHANGEDTODS$YEAR)*# disperion parameter for overdispersed models*

mod4.a<-**glmer**(COUNTRAW ~ **as.factor**(YEAR) + SOCIAL.STATUS  + (1|WEEK) +  (1|MALE.ID) +(1|dispersionDS),data=CHANGEDTODS,family="poisson")*#model overdispersed so added dispersion parameter*
**summary**(mod4.a)

## Generalized linear mixed model fit by maximum likelihood (Laplace
##   Approximation) [glmerMod]
##  Family: poisson  ( log )
## Formula: COUNTRAW ~ as.factor(YEAR) + SOCIAL.STATUS + (1 | WEEK) + ## (1 | MALE.ID) + (1 | dispersionDS)
##    Data: CHANGEDTODS
##
##      AIC      BIC   logLik deviance df.resid
##    257.7    264.7   -121.8    243.7       13
##
## Scaled residuals:
##      Min       1Q   Median       3Q      Max
## -0.29009 -0.18281 -0.00837  0.11728  0.32824
##
## Random effects:
##  Groups       Name        Variance Std.Dev.
##  dispersionDS (Intercept) 0.075765 0.27525
##  MALE.ID      (Intercept) 0.027658 0.16631
##  WEEK         (Intercept) 0.003024 0.05499
## Number of obs: 20, groups:  dispersionDS, 20; MALE.ID, 10; WEEK, 5
##
## Fixed effects:
##                     Estimate Std. Error z value Pr(>|z|)    
## (Intercept)          6.06827    0.25941  23.393   <2e-16 ***
## as.factor(YEAR)2014 -0.52755    0.27652  -1.908   0.0564 .  
## as.factor(YEAR)2015  0.13801    0.29012   0.476   0.6343    
## SOCIAL.STATUSDS     -0.09749    0.12576  -0.775   0.4382    
## ---
## Signif. codes:  0 '***' 0.001 '**' 0.01 '*' 0.05 '.' 0.1 ' ' 1
##
## Correlation of Fixed Effects:
##              (Intr) a.(YEAR)2014 a.(YEAR)2015
## a.(YEAR)2014 -0.876                          
## a.(YEAR)2015 -0.826  0.776                   
## SOCIAL.STAT  -0.237 -0.006       -0.004

**overdisp.glmer**(mod4.a)*# check for over/under-dispersion*

## Residual deviance: 0.604 on 13 degrees of freedom (ratio: 0.046)

**confint**(mod4.a, level=0.95, method="Wald",oldNames=F) *#generate 95%CI using Wald method*

##                                  2.5 %     97.5 %
## sd_(Intercept)|dispersionDS         NA         NA
## sd_(Intercept)|MALE.ID              NA         NA
## sd_(Intercept)|WEEK                 NA         NA
## (Intercept)                  5.5598439 6.57669612
## as.factor(YEAR)2014         -1.0695157 0.01441253
## as.factor(YEAR)2015         -0.4306062 0.70663518
## SOCIAL.STATUSDS             -0.3439648 0.14898771

*#Test for significance of random predictor male ID*
mod4.1<-**glmer**(COUNTRAW ~ **as.factor**(YEAR) + SOCIAL.STATUS  + (1|WEEK)+(1|dispersionDS) ,data=CHANGEDTODS,family="poisson")
**anova**(mod4.a,mod4.1)

## Data: CHANGEDTODS
## Models:
## mod4.1: COUNTRAW ~ as.factor(YEAR) + SOCIAL.STATUS + (1 | WEEK) + (1 |
## mod4.1:     dispersionDS)
## mod4.a: COUNTRAW ~ as.factor(YEAR) + SOCIAL.STATUS + (1 | WEEK) + (1 |
## mod4.a:     MALE.ID) + (1 | dispersionDS)
##        Df    AIC    BIC  logLik deviance  Chisq Chi Df Pr(>Chisq)
## mod4.1  6 256.18 262.15 -122.09   244.18                         
## mod4.a  7 257.69 264.66 -121.84   243.69 0.4887      1     0.4845

Models that compare sperm counts for males that are D(stage 1) that become DD (stage 2).

CHANGEDTODD<-**read.table**(file=" CHANGE_COUNT_D_TO_DD.csv",header=T,row.names=NULL,sep=",")*#load data frame*

mod5<-**glmer**(COUNTRAW ~ **as.factor**(YEAR) + SOCIAL.STATUS  + (1|WEEK) +  (1|MALE.ID) ,data=CHANGEDTODD,family="poisson")
**overdisp.glmer**(mod5)*# check for over/under-dispersion*

## Residual deviance: 228.037 on 14 degrees of freedom (ratio: 16.288)

dispersionDD<-1:**length**(CHANGEDTODD$YEAR)*# disperion parameter for overdispersed models*

mod5.a<-**glmer**(COUNTRAW ~ **as.factor**(YEAR) + SOCIAL.STATUS  + (1|WEEK) +  (1|MALE.ID) +(1|dispersionDD),data=CHANGEDTODD,family="poisson")*#model overdispersed so added dispersion parameter*
**summary**(mod5.a)

## Generalized linear mixed model fit by maximum likelihood (Laplace
##   Approximation) [glmerMod]
##  Family: poisson  ( log )
## Formula: COUNTRAW ~ as.factor(YEAR) + SOCIAL.STATUS + (1 | WEEK) + ## (1 | MALE.ID) + (1 | dispersionDD)
##    Data: CHANGEDTODD
##
##      AIC      BIC   logLik deviance df.resid
##    247.3    254.3   -116.7    233.3       13
##
## Scaled residuals:
##      Min       1Q   Median       3Q      Max
## -0.61632 -0.11273  0.03231  0.10608  0.27940
##
## Random effects:
##  Groups       Name        Variance  Std.Dev.
##  dispersionDD (Intercept) 6.514e-02 2.552e-01
##  MALE.ID      (Intercept) 4.505e-09 6.712e-05
##  WEEK         (Intercept) 2.874e-03 5.361e-02
## Number of obs: 20, groups:  dispersionDD, 20; MALE.ID, 10; WEEK, 5
##
## Fixed effects:
##                     Estimate Std. Error z value Pr(>|z|)    
## (Intercept)           5.6523     0.1598   35.37   <2e-16 ***
## as.factor(YEAR)2014   0.2112     0.1681    1.26    0.209    
## as.factor(YEAR)2015   0.1888     0.1953    0.97    0.334    
## SOCIAL.STATUSDD      -0.1647     0.1170   -1.41    0.159    
## ---
## Signif. codes:  0 '***' 0.001 '**' 0.01 '*' 0.05 '.' 0.1 ' ' 1
##
## Correlation of Fixed Effects:
##              (Intr) a.(YEAR)2014 a.(YEAR)2015
## a.(YEAR)2014 -0.800                          
## a.(YEAR)2015 -0.753  0.713                   
## SOCIAL.STAT  -0.368  0.002        0.005

**confint**(mod5.a, level=0.95, method="Wald",oldNames=F) *#generate 95%CI using Wald method*

##                                  2.5 %     97.5 %
## sd_(Intercept)|dispersionDD         NA         NA
## sd_(Intercept)|MALE.ID              NA         NA
## sd_(Intercept)|WEEK                 NA         NA
## (Intercept)                  5.3390749 5.96545984
## as.factor(YEAR)2014         -0.1182952 0.54076135
## as.factor(YEAR)2015         -0.1940943 0.57161437
## SOCIAL.STATUSDD             -0.3940623 0.06472596

*#Test for significance of random predictor male ID*
mod5.1<-**glmer**(COUNTRAW ~ **as.factor**(YEAR) + SOCIAL.STATUS  + (1|WEEK)+(1|dispersionDD) ,data=CHANGEDTODD,family="poisson")
**anova**(mod5.a,mod5.1)

## Data: CHANGEDTODD
## Models:
## mod5.1: COUNTRAW ~ as.factor(YEAR) + SOCIAL.STATUS + (1 | WEEK) + (1 |
## mod5.1:     dispersionDD)
## mod5.a: COUNTRAW ~ as.factor(YEAR) + SOCIAL.STATUS + (1 | WEEK) + (1 |
## mod5.a:     MALE.ID) + (1 | dispersionDD)
##        Df    AIC    BIC  logLik deviance Chisq Chi Df Pr(>Chisq)
## mod5.1  6 245.33 251.31 -116.67   233.33                        
## mod5.a  7 247.33 254.30 -116.67   233.33     0      1          1

Models that compare sperm counts for males that are S (stage 1) that become SD (stage 2).

CHANGESTOSD<-**read.table**(file=" CHANGE_COUNT_S_TO_SD.csv",header=T,row.names=NULL,sep=",")*#load data frame*

mod6<-**glmer**(COUNTRAW ~ **as.factor**(YEAR) + SOCIAL.STATUS  + (1|WEEK) +  (1|MALE.ID) ,data=CHANGESTOSD,family="poisson")
**overdisp.glmer**(mod6)*# check for over/under-dispersion*

## Residual deviance: 435.473 on 12 degrees of freedom (ratio: 36.289)

dispersionSD<-1:**length**(CHANGESTOSD$YEAR)*# disperion parameter for overdispersed models*

mod6.a<-**glmer**(COUNTRAW ~ **as.factor**(YEAR) + SOCIAL.STATUS  + (1|WEEK) +  (1|MALE.ID)+(1|dispersionSD),data=CHANGESTOSD,family="poisson")*#model overdispersed so added dispersion parameter*
**summary**(mod6.a)

## Generalized linear mixed model fit by maximum likelihood (Laplace
##   Approximation) [glmerMod]
##  Family: poisson  ( log )
## Formula: COUNTRAW ~ as.factor(YEAR) + SOCIAL.STATUS + (1 | WEEK) + ## (1 | MALE.ID) + (1 | dispersionSD)
##    Data: CHANGESTOSD
##
##      AIC      BIC   logLik deviance df.resid
##    234.5    240.7   -110.2    220.5       11
##
## Scaled residuals:
##      Min       1Q   Median       3Q      Max
## -0.36301 -0.10226  0.00885  0.07905  0.30047
##
## Random effects:
##  Groups       Name        Variance  Std.Dev.
##  dispersionSD (Intercept) 6.485e-02 2.547e-01
##  MALE.ID      (Intercept) 1.024e-10 1.012e-05
##  WEEK         (Intercept) 2.426e-03 4.925e-02
## Number of obs: 18, groups:  dispersionSD, 18; MALE.ID, 9; WEEK, 5
##
## Fixed effects:
##                     Estimate Std. Error z value Pr(>|z|)    
## (Intercept)           6.4345     0.1256   51.23  < 2e-16 ***
## as.factor(YEAR)2014  -0.5832     0.1519   -3.84 0.000124 ***
## as.factor(YEAR)2015  -0.2383     0.1515   -1.57 0.115878    
## SOCIAL.STATUSSD      -0.2379     0.1224   -1.94 0.051876 .  
## ---
## Signif. codes:  0 '***' 0.001 '**' 0.01 '*' 0.05 '.' 0.1 ' ' 1
##
## Correlation of Fixed Effects:
##              (Intr) a.(YEAR)2014 a.(YEAR)2015
## a.(YEAR)2014 -0.594                          
## a.(YEAR)2015 -0.605  0.490                   
## SOCIAL.STAT  -0.487  0.005        0.001

**overdisp.glmer**(mod6.a)*# check for over/under-dispersion*

## Residual deviance: 0.605 on 11 degrees of freedom (ratio: 0.055)

**confint**(mod6.a, level=0.95, method="Wald",oldNames=F) *#generate 95%CI using Wald method*

##                                  2.5 %       97.5 %
## sd_(Intercept)|dispersionSD         NA           NA
## sd_(Intercept)|MALE.ID              NA           NA
## sd_(Intercept)|WEEK                 NA           NA
## (Intercept)                  6.1882898  6.680617265
## as.factor(YEAR)2014         -0.8809774 -0.285446552
## as.factor(YEAR)2015         -0.5352977  0.058746261
## SOCIAL.STATUSSD             -0.4777961  0.001933525

*#Test for significance of random predictor male ID*
mod6.1<-**glmer**(COUNTRAW ~ **as.factor**(YEAR) + SOCIAL.STATUS  + +(1|dispersionSD)+(1|WEEK) ,data=CHANGESTOSD,family="poisson")
**anova**(mod6.a,mod6.1)

## Data: CHANGESTOSD
## Models:
## mod6.1: COUNTRAW ~ as.factor(YEAR) + SOCIAL.STATUS + +(1 | dispersionSD) +
## mod6.1:     (1 | WEEK)
## mod6.a: COUNTRAW ~ as.factor(YEAR) + SOCIAL.STATUS + (1 | WEEK) + (1 |
## mod6.a:     MALE.ID) + (1 | dispersionSD)
##        Df    AIC    BIC  logLik deviance Chisq Chi Df Pr(>Chisq)
## mod6.1  6 232.47 237.81 -110.23   220.47                        
## mod6.a  7 234.47 240.70 -110.23   220.47     0      1          1

Models that compare sperm counts for males that are S (stage 1) that become SS (stage 2).

CHANGESTOSS<-**read.table**(file=" CHANGE_COUNT_S_TO_SS.csv",header=T,row.names=NULL,sep=",")*#load data frame*

mod7<-**glmer**(COUNTRAW ~ **as.factor**(YEAR) + SOCIAL.STATUS  + (1|WEEK) +  (1|MALE.ID) ,data=CHANGESTOSS,family="poisson")
**overdisp.glmer**(mod7)*# check for over/under-dispersion*

## Residual deviance: 406.822 on 12 degrees of freedom (ratio: 33.902)

dispersionSS<-1:**length**(CHANGESTOSS$YEAR)*# disperion parameter for overdispersed models*

mod7.a<-**glmer**(COUNTRAW ~ **as.factor**(YEAR) + SOCIAL.STATUS  + (1|WEEK) +  (1|MALE.ID) + (1|dispersionSS),data=CHANGESTOSS,family="poisson")*#model overdispersed so added dispersion parameter*
**summary**(mod7.a)

## Generalized linear mixed model fit by maximum likelihood (Laplace
##   Approximation) [glmerMod]
##  Family: poisson  ( log )
## Formula: COUNTRAW ~ as.factor(YEAR) + SOCIAL.STATUS + (1 | WEEK) + (1 |  
##     MALE.ID) + (1 | dispersionSS)
##    Data: CHANGESTOSS
##
##      AIC      BIC   logLik deviance df.resid
##    238.2    244.4   -112.1    224.2       11
##
## Scaled residuals:
##      Min       1Q   Median       3Q      Max
## -0.31801 -0.14250 -0.03193  0.05426  0.25890
##
## Random effects:
##  Groups       Name        Variance  Std.Dev.
##  dispersionSS (Intercept) 7.771e-02 2.788e-01
##  MALE.ID      (Intercept) 0.000e+00 0.000e+00
##  WEEK         (Intercept) 3.521e-10 1.876e-05
## Number of obs: 18, groups:  dispersionSS, 18; MALE.ID, 10; WEEK, 5
##
## Fixed effects:
##                     Estimate Std. Error z value Pr(>|z|)    
## (Intercept)          6.35425    0.13301   47.77  < 2e-16 ***
## as.factor(YEAR)2014 -0.58610    0.16343   -3.59 0.000335 ***
## as.factor(YEAR)2015 -0.39093    0.16313   -2.40 0.016557 *  
## SOCIAL.STATUSSS      0.09117    0.13346    0.68 0.494526    
## ---
## Signif. codes:  0 '***' 0.001 '**' 0.01 '*' 0.05 '.' 0.1 ' ' 1
##
## Correlation of Fixed Effects:
##              (Intr) a.(YEAR)2014 a.(YEAR)2015
## a.(YEAR)2014 -0.608                          
## a.(YEAR)2015 -0.611  0.497                   
## SOCIAL.STAT  -0.501 -0.003        0.000

**confint**(mod7.a, level=0.95, method="Wald",oldNames=F) *#generate 95%CI using Wald method*

##                                  2.5 %      97.5 %
## sd_(Intercept)|dispersionSS         NA          NA
## sd_(Intercept)|MALE.ID              NA          NA
## sd_(Intercept)|WEEK                 NA          NA
## (Intercept)                  6.0935652  6.61494461
## as.factor(YEAR)2014         -0.9064065 -0.26578688
## as.factor(YEAR)2015         -0.7106708 -0.07119744
## SOCIAL.STATUSSS             -0.1704098  0.35275361

*#Test for significance of random predictor male ID*
mod7.1<-**glmer**(COUNTRAW ~ **as.factor**(YEAR) + SOCIAL.STATUS  + (1|WEEK)+ (1|dispersionSS) ,data=CHANGESTOSS,family="poisson")
**anova**(mod7.a,mod7.1)

## Data: CHANGESTOSS
## Models:
## mod7.1: COUNTRAW ~ as.factor(YEAR) + SOCIAL.STATUS + (1 | WEEK) + (1 |
## mod7.1:     dispersionSS)
## mod7.a: COUNTRAW ~ as.factor(YEAR) + SOCIAL.STATUS + (1 | WEEK) + (1 |
## mod7.a:     MALE.ID) + (1 | dispersionSS)
##        Df    AIC    BIC logLik deviance Chisq Chi Df Pr(>Chisq)
## mod7.1  6 236.21 241.55 -112.1   224.21                        
## mod7.a  7 238.21 244.44 -112.1   224.21     0      1     0.9998

Model that tests for an interaction effect between social phenotype and experimental stage with VAP as response variable.

TESTFORINTERACTIONVAP<-read.table(file="CHANGESSSPLOT.csv",header=T,row.names=NULL,sep=",")#load data frame

modelINT<-lmer(VAP ~ as.factor(YEAR) + GROUP + as.factor(STAGE) + GROUP:as.factor(STAGE) + (1|WEEK) + (1|MALE.ID) ,data=TESTFORINTERACTIONVAP)
summary(modelINT)

## Linear mixed model fit by REML t-tests use Satterthwaite approximations
## to degrees of freedom [lmerMod]
## Formula:
## VAP ~ as.factor(YEAR) + GROUP + as.factor(STAGE) + GROUP:as.factor(STAGE) +
## (1 | WEEK) + (1 | MALE.ID)
## Data: TESTFORINTERACTIONVAP
##
## REML criterion at convergence: 1302.9
##
## Scaled residuals:
## Min 1Q Median 3Q Max
## -2.52976 -0.59331 -0.08737 0.65619 2.01827
##
## Random effects:
## Groups Name Variance Std.Dev.
## MALE.ID (Intercept) 354.15 18.819
## WEEK (Intercept) 83.96 9.163
## Residual 325.72 18.048
## Number of obs: 151, groups: MALE.ID, 38; WEEK, 5
##
## Fixed effects:
## Estimate Std. Error df t value Pr(>|t|)
## (Intercept) 142.0250 10.7297 23.3900 13.237 2.36e-12 ***
## as.factor(YEAR)2014 22.5507 9.0120 31.5900 2.502 0.0177 *
## as.factor(YEAR)2015 15.9919 9.5815 31.5600 1.669 0.1050
## GROUPD-S -1.8400 10.1686 42.4800 -0.181 0.8573
## GROUPS-D 1.2965 10.5956 42.6500 0.122 0.9032
## GROUPS-S 12.2020 10.5956 42.6500 1.152 0.2559
## as.factor(STAGE)2 -0.2957 5.7216 108.7500 -0.052 0.9589
## GROUPD-S: (STAGE)2 18.9140 8.1476 108.3200 2.321 0.0221 *
## GROUPS-D: (STAGE)2 -8.6932 8.3023 108.1200 -1.047 0.2974
## GROUPS-S: (STAGE)2 -0.8765 8.3023 108.1200 -0.106 0.9161
## ---
## Signif. codes: 0 '***' 0.001 '**' 0.01 '*' 0.05 '.' 0.1 ' ' 1
##
## Correlation of Fixed Effects:
## (Intr) a.(YEAR)2014 a.(YEAR)2015 GROUPD-S GROUPS-D GROUPS-S
## a.(YEAR)2014 -0.597
## a.(YEAR)2015 -0.519 0.592
## GROUPD-S -0.474 0.000 0.000
## GROUPS-D -0.528 0.132 0.059 0.480
## GROUPS-S -0.528 0.132 0.059 0.480 0.488
## as.(STAGE)2 -0.275 0.012 0.018 0.280 0.275 0.275
## GROUPD-S:.( 0.192 -0.005 -0.015 -0.393 -0.192 -0.192
## GROUPS-D:.( 0.190 -0.008 -0.012 -0.193 -0.396 -0.190
## GROUPS-S:.( 0.190 -0.008 -0.012 -0.193 -0.190 -0.396
## a.(STA GROUPD-S: GROUPS-D:
## a.(YEAR)2014
## a.(YEAR)2015
## GROUPD-S
## GROUPS-D
## GROUPS-S
## as.(STAGE)2
## GROUPD-S:.( -0.702
## GROUPS-D:.( -0.689 0.484
## GROUPS-S:.( -0.689 0.484 0.475

confint(modelINT, level=0.95, method="Wald",oldNames=F) #generate 95%CI using Wald method

## 2.5 % 97.5 %
## sd_(Intercept)|MALE.ID NA NA
## sd_(Intercept)|WEEK NA NA
## sigma NA NA
## (Intercept) 120.995106 163.054952
## as.factor(YEAR)2014 4.887503 40.213940
## as.factor(YEAR)2015 -2.787519 34.771282
## GROUPD-S -21.770180 18.090180
## GROUPS-D -19.470444 22.063425
## GROUPS-S -8.564888 32.968981
## as.factor(STAGE)2 -11.509833 10.918476
## GROUPD-S:as.factor(STAGE)2 2.945134 34.882962
## GROUPS-D:as.factor(STAGE)2 -24.965396 7.578975
## GROUPS-S:as.factor(STAGE)2 -17.148729 15.395642


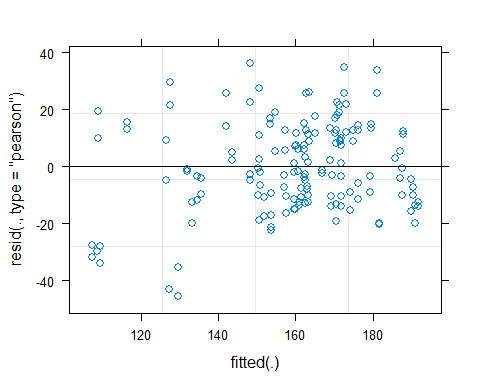
plot(modelINT, results="hide", fig.show='hide') #plot residuals vs fitted values to check for unequal variance

qqmath(modelINT) #check normality assumption
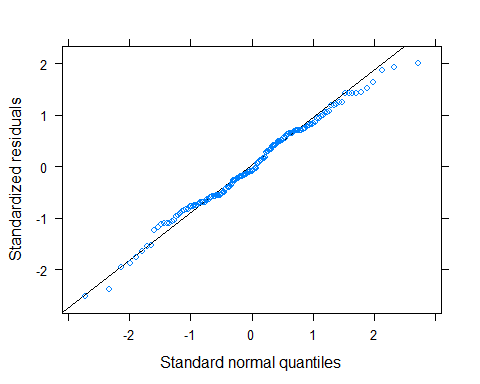


shapiro.test(resid(modelINT))
## Shapiro-Wilk normality test
## data: resid(modelINT)
## W = 0.99013, p-value = 0.3721

#Test for significance of random predictor male ID
modelINT.1<-lmer(VAP ~ as.factor(YEAR) + GROUP*as.factor(STAGE) + (1|WEEK) ,data=TESTFORINTERACTIONVAP)
anova(modelINT,modelINT.1)

## refitting model(s) with ML (instead of REML)

## Data: TESTFORINTERACTIONVAP
## Models:
## ..1: VAP ~ as.factor(YEAR) + GROUP * as.factor(STAGE) + (1 | WEEK)
## object: VAP ~ as.factor(YEAR) + GROUP + as.factor(STAGE) + GROUP:as.factor(STAGE) +
## object: (1 | WEEK) + (1 | MALE.ID)
## Df AIC BIC logLik deviance Chisq Chi Df Pr(>Chisq)
## ..1 12 1422.7 1458.9 -699.37 1398.7
## object 13 1385.5 1424.8 -679.76 1359.5 39.212 1 3.802e-10 ***
## ---
## Signif. codes: 0 '***' 0.001 '**' 0.01 '*' 0.05 '.' 0.1 ' ' 1

#Test for significance of overall interaction term
modelINT.2<-lmer(VAP ~ as.factor(YEAR) + GROUP + as.factor(STAGE) + (1|WEEK)+ (1|MALE.ID) ,data=TESTFORINTERACTIONVAP)
anova(modelINT,modelINT.2)

## refitting model(s) with ML (instead of REML)

## Data: TESTFORINTERACTIONVAP
## Models:
## ..1: VAP ~ as.factor(YEAR) + GROUP + as.factor(STAGE) + (1 | WEEK) +
## ..1: (1 | MALE.ID)
## object: VAP ~ as.factor(YEAR) + GROUP + as.factor(STAGE) + GROUP:as.factor(STAGE) +
## object: (1 | WEEK) + (1 | MALE.ID)
## Df AIC BIC logLik deviance Chisq Chi Df Pr(>Chisq)
## ..1 10 1391.3 1421.5 -685.67 1371.3
## object 13 1385.5 1424.8 -679.76 1359.5 11.806 3 0.008077 **
## ---
## Signif. codes: 0 '***' 0.001 '**' 0.01 '*' 0.05 '.' 0.1 ' ' 1

Model that tests for an interaction effect between social phenotype and experimental stage with sperm concentration as response variable.

TESTFORINTERACTIONCOUNT<-read.table(file="CHANGESSPLOT2.csv",header=T,row.names=NULL,sep=",")#load data frame
modelINT2<-glmer(COUNTB ~ as.factor(YEAR) + GROUP*as.factor(STAGE) +(1|WEEK)+ (1|MALE.ID) ,data=TESTFORINTERACTIONCOUNT, family="poisson")
overdisp.glmer(modelINT2)# check for over/under-dispersion

## Residual deviance: 1337.064 on 64 degrees of freedom (ratio: 20.892)

#Model failed to converge with the addition of dispersion parameter so removing non-significant random term (1|WEEK)

#Test for significance of random predictor WEEK
modelINT2.1<-glmer(COUNTB ~ as.factor(YEAR) + GROUP*as.factor(STAGE) + (1|MALE.ID) ,data=TESTFORINTERACTIONCOUNT, family="poisson")
anova(modelINT2,modelINT2.1)

## Data: TESTFORINTERACTIONCOUNT
## Models:
## modelINT2.1: COUNTB ~ as.factor(YEAR) + GROUP * as.factor(STAGE) + (1 | MALE.ID)
## modelINT2: COUNTB ~ as.factor(YEAR) + GROUP * as.factor(STAGE) + (1 | WEEK) +
## modelINT2: (1 | MALE.ID)
## Df AIC BIC logLik deviance Chisq Chi Df Pr(>Chisq)
## modelINT2.1 11 2140.8 2166.4 -1059.4 2118.8
## modelINT2 12 2142.8 2170.8 -1059.4 2118.8 0 1 1

dispersionINT2<-1:length(TESTFORINTERACTIONCOUNT$YEAR)# dispersion parameter for overdispersed model

#running model with dispersion parameter and (1|WEEK) removed
modINT2.2<-glmer(COUNTB ~ as.factor(YEAR) + GROUP*as.factor(STAGE) + (1|MALE.ID) +(1|dispersionINT2),data=TESTFORINTERACTIONCOUNT,family="poisson")#model overdispersed so added dispersion parameter
overdisp.glmer(modINT2.2)# check for over/under-dispersion

## Residual deviance: 2.077 on 64 degrees of freedom (ratio: 0.032)

summary(modINT2.2)

## Generalized linear mixed model fit by maximum likelihood (Laplace
## Approximation) [glmerMod]
## Family: poisson ( log )
## Formula:
## COUNTB ~ as.factor(YEAR) + GROUP * as.factor(STAGE) + (1 | MALE.ID) +
## (1 | dispersionINT2)
## Data: TESTFORINTERACTIONCOUNT
##
## AIC BIC logLik deviance df.resid
## 967.4 995.4 -471.7 943.4 64
##
## Scaled residuals:
## Min 1Q Median 3Q Max
## -0.39724 -0.13159 -0.00873 0.10502 0.25812
##
## Random effects:
## Groups Name Variance Std.Dev.
## dispersionINT2 (Intercept) 0.08549 0.2924
## MALE.ID (Intercept) 0.02114 0.1454
## Number of obs: 76, groups: dispersionINT2, 76; MALE.ID, 38
##
## Fixed effects:
## Estimate Std. Error z value Pr(>|z|)
## (Intercept) 6.04682 0.12989 46.55 < 2e-16 ***
## as.factor(YEAR)2014 -0.39715 0.10511 -3.78 0.000158 ***
## as.factor(YEAR)2015 -0.11287 0.10997 -1.03 0.304735
## GROUPD-S 0.03616 0.14820 0.24 0.807235
## GROUPS-D 0.27538 0.15256 1.81 0.071053 .
## GROUPS-S 0.14945 0.15239 0.98 0.326723
## as.factor(STAGE)2 -0.16450 0.13331 -1.23 0.217202
## GROUPD-S: (STAGE)2 0.06689 0.18849 0.35 0.722700
## GROUPS-D: (STAGE)2 -0.07374 0.19322 -0.38 0.702732
## GROUPS-S: (STAGE)2 0.26365 0.19427 1.36 0.174727
## ---
## Signif. codes: 0 '***' 0.001 '**' 0.01 '*' 0.05 '.' 0.1 ' ' 1
##
## Correlation of Fixed Effects:
## (Intr) a.(YEAR)2014 a.(YEAR)2015 GROUPD-S GROUPS-D GROUPS-S
## a.(YEAR)2014 -0.554
## a.(YEAR)2015 -0.492 0.584
## GROUPD-S -0.572 0.003 0.001
## GROUPS-D -0.602 0.095 0.038 0.486
## GROUPS-S -0.605 0.099 0.041 0.486 0.494
## as.(STAGE)2 -0.512 0.000 0.002 0.449 0.436 0.436
## GROUPD-S:.( 0.363 -0.002 -0.002 -0.635 -0.308 -0.309
## GROUPS-D:.( 0.353 0.001 -0.001 -0.309 -0.631 -0.301
## GROUPS-S:.( 0.346 0.008 0.007 -0.308 -0.307 -0.634
## a.(STA GROUPD-S: GROUPS-D:
## a.(YEAR)2014
## a.(YEAR)2015
## GROUPD-S
## GROUPS-D
## GROUPS-S
## as.(STAGE)2
## GROUPD-S:.( -0.707
## GROUPS-D:.( -0.690 0.488
## GROUPS-S:.( -0.686 0.485 0.473

confint(modINT2.2,level=0.95, method="Wald",oldNames=F) #generate 95%CI using Wald method

## 2.5 % 97.5 %
## sd_(Intercept)|dispersionINT2 NA NA
## sd_(Intercept)|MALE.ID NA NA
## (Intercept) 5.79224548 6.30140126
## as.factor(YEAR)2014 -0.60316188 -0.19114440
## as.factor(YEAR)2015 -0.32841239 0.10267428
## GROUPD-S -0.25429946 0.32661757
## GROUPS-D -0.02361979 0.57438865
## GROUPS-S -0.14922277 0.44812744
## as.factor(STAGE)2 -0.42577221 0.09677437
## GROUPD-S:as.factor(STAGE)2 -0.30254368 0.43631449
## GROUPS-D:as.factor(STAGE)2 -0.45244547 0.30496596
## GROUPS-S:as.factor(STAGE)2 -0.11710279 0.64440879

#Test for significance of overall interaction effect
modINT2.3<-glmer(COUNTB ~ as.factor(YEAR) + GROUP + as.factor(STAGE) + (1|MALE.ID) +(1|dispersionINT2) ,data=TESTFORINTERACTIONCOUNT, family="poisson")
anova(modINT2.2,modINT2.3)

## Data: TESTFORINTERACTIONCOUNT
## Models:
## modINT2.3: COUNTB ~ as.factor(YEAR) + GROUP + as.factor(STAGE) + (1 | MALE.ID) +
## modINT2.3: (1 | dispersionINT2)
## modINT2.2: COUNTB ~ as.factor(YEAR) + GROUP * as.factor(STAGE) + (1 | MALE.ID) +
## modINT2.2: (1 | dispersionINT2)
## Df AIC BIC logLik deviance Chisq Chi Df Pr(>Chisq)
## modINT2.3 9 964.46 985.44 -473.23 946.46
## modINT2.2 12 967.41 995.38 -471.71 943.41 3.044 3 0.3849

#Test for significance of Male ID
modINT2.4<-glmer(COUNTB ~ as.factor(YEAR) + GROUP*as.factor(STAGE) +(1|dispersionINT2) ,data=TESTFORINTERACTIONCOUNT, family="poisson")
anova(modINT2.2,modINT2.4)

## Data: TESTFORINTERACTIONCOUNT
## Models:
## modINT2.4: COUNTB ~ as.factor(YEAR) + GROUP * as.factor(STAGE) + (1 | dispersionINT2)
## modINT2.2: COUNTB ~ as.factor(YEAR) + GROUP * as.factor(STAGE) + (1 | MALE.ID) +
## modINT2.2: (1 | dispersionINT2)
## Df AIC BIC logLik deviance Chisq Chi Df Pr(>Chisq)
## modINT2.4 11 966.79 992.43 -472.39 944.79
## modINT2.2 12 967.41 995.38 -471.71 943.41 1.3743 1 0.2411

**Seminal Fluid effect on sperm velocity**

>SEMINALFLUIDSWAP<-read.table(file="SEMINAL_FLUID_SWAP.csv",header=T,row.names=NULL,sep=",")#import data frame

> #Social status as a fixed effect

> modswap<-lmer(Difference.in.rivals.SF ~ as.factor(YEAR) +SOCIAL.STATUS.SF + (1|pair) +(1|WEEK)+(1|Male.ID.sperm)+(1|Rivals.ID),data=SEMINALFLUIDSWAP)
> summary(modswap)

## Linear mixed model fit by REML
## t-tests use  Satterthwaite approximations to degrees of freedom

## ['lmerMod']
## Formula: Difference.in.rivals.SF ~ as.factor(YEAR) + SOCIAL.STATUS.SF + (1 | pair) ## + (1 | WEEK) + (1 | Male.ID.sperm) + (1 | Rivals.ID)
##  Data: SEMINALFLUIDSWAP
##
## REML criterion at convergence: 756
##
## Scaled residuals:
##     Min       1Q   Median       3Q      Max
## -2.92965 -0.52134  0.06241  0.67950  2.06092
##
## Random effects:
## Groups        Name        Variance  Std.Dev.
## Rivals.ID     (Intercept) 1.192e-15 3.452e-08
## Male.ID.sperm (Intercept) 0.000e+00 0.000e+00
## pair          (Intercept) 0.000e+00 0.000e+00
## WEEK          (Intercept) 0.000e+00 0.000e+00
## Residual                  1.348e+03 3.672e+01
## Number of obs: 78, groups:  Rivals.ID, 42; Male.ID.sperm, 42; pair, 39; ## WEEK, 5
##
## Fixed effects:
##                    Estimate Std. Error      df t value Pr(>|t|)    
## (Intercept)          -24.387      8.863  74.000  -2.752 0.007455 **
## as.factor(YEAR)2014    3.549     10.168  74.000   0.349 0.728053    
## as.factor(YEAR)2015    5.793     10.837  74.000   0.535 0.594545    
## SS.SFSubdominant      31.421      8.314  74.000   3.779 0.000316 ***
## ---
## Signif. codes:  0 ‘***’ 0.001 ‘**’ 0.01 ‘*’ 0.05 ‘.’ 0.1 ‘ ’ 1
##
## Correlation of Fixed Effects:
##             (Intr) a.(YEAR)2014 a.(YEAR)2015
## a.(YEAR)2014 -0.680                          
## a.(YEAR)2015 -0.638  0.556                   
## SS.SFSbdmnn  -0.469  0.000        0.000      
> confint(modswap, level=0.95, method="Wald",oldNames=F) #generate 95%CI using Wald method
##                                  2.5 %    97.5 %
## (Intercept)                     -41.75825   -7.015507
## as.factor(YEAR)2014             -16.38065 23.478988
## as.factor(YEAR)2015            -15.44683   27.033191
## SS.SFSubdominant                15.12494   47.716517

> qqmath(modswap)


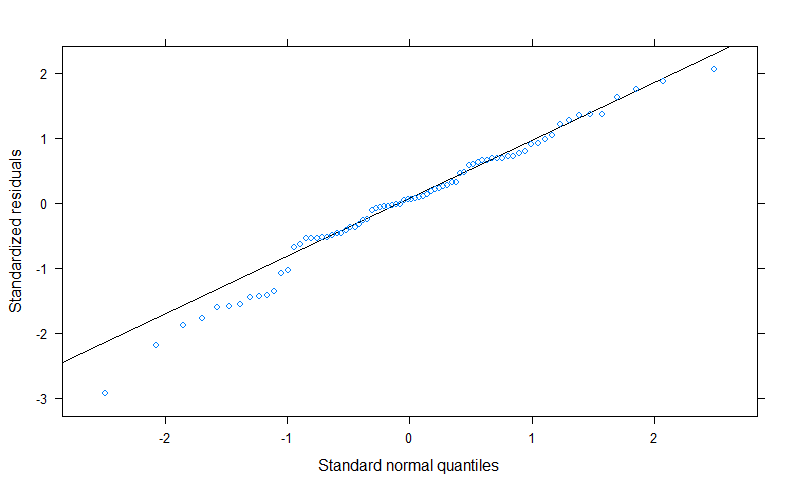


> shapiro.test(resid(modswap))

## Shapiro-Wilk normality test
##
## data:  resid(modswap)
## W = 0.97921, p-value = 0.2321

> plot(modswap, results="hide", fig.show='hide')


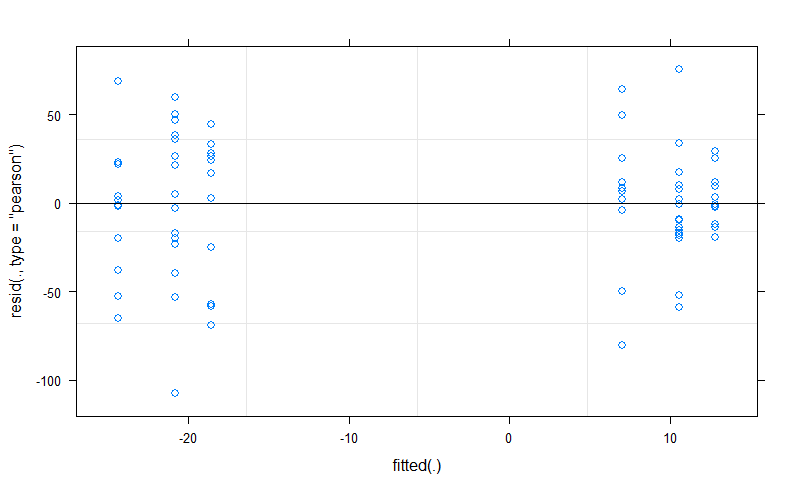


Now add Difference in control speeds as a fixed effect

> modswapB<-lmer(Difference.in.rivals.SF ~ as.factor(YEAR) +SOCIAL.STATUS.SF + Difference.own.speeds  + (1|pair) +(1|WEEK) +(1|Male.ID.sperm) +(1|Rivals.ID),data=SEMINALFLUIDSWAP)
> summary(modswapB)

## Linear mixed model fit by REML
## t-tests use  Satterthwaite approximations to degrees of freedom

##  ['lmerMod']
## Formula: Difference.in.rivals.SF ~ as.factor(YEAR) + SOCIAL.STATUS.SF +

## Difference.own.speeds +  
##    (1 | pair) + (1 | WEEK) + (1 | Male.ID.sperm) + (1 | Rivals.ID)
##   Data: SEMINALFLUIDSWAP
##
## REML criterion at convergence: 720.7
##
## Scaled residuals:
##    Min      1Q  Median      3Q     Max
## -2.4029 -0.3984  0.1413  0.5410  1.4050
##
## Random effects:
## Groups        Name        Variance Std.Dev.
## Rivals.ID     (Intercept) 192.62   13.879  
## Male.ID.sperm (Intercept)  60.82    7.799  
## pair          (Intercept)   0.00    0.000  
## WEEK          (Intercept)   0.00    0.000  
## Residual                  595.21   24.397  
## Number of obs: 78, groups:  Rivals.ID, 42; Male.ID.sperm, 42; pair, 39; ## WEEK, 5
##
## Fixed effects:
##                       Estimate Std. Error    df t value Pr(>|t|)    
## (Intercept)           -12.474     7.909   34.640  1.577    0.124    
## as.factor(YEAR)2014     2.11001    9.06235  28.570   0.233    0.818    
## as.factor(YEAR)2015     5.26272    9.73634  26.650   0.541    0.593    
## SS.SFSubdominant        8.65605    7.39091  64.100   1.171    0.246    
## Difference.own.speeds   0.72143    0.09715  69.020   7.426 2.24e-10 ***
## ---
## Signif. codes:  0 ‘***’ 0.001 ‘**’ 0.01 ‘*’ 0.05 ‘.’ 0.1 ‘ ’ 1
##
## Correlation of Fixed Effects:
##            (Intr) a.(YEAR)2014 a.(YEAR)2015 SS.SFS
## a.(YEAR)2014 -0.681                                 
## a.(YEAR)2015 -0.635  0.554                          
## SS.SFSbdmnn  -0.468 -0.002        0.000             
## Dffrnc.wn.s   0.223 -0.001       -0.001       -0.475
> confint(modswapB, level=0.95, method="Wald",oldNames=F) #generate 95%CI using Wald method
##                                   2.5 %     97.5 %
## (Intercept)                  -27.9773639  3.0292051
## as.factor(YEAR)2014          -15.6518711 19.8718874
## as.factor(YEAR)2015          -13.8201595 24.3456017
## SS.SFSubdominant              -5.8298735 23.1419734
## Difference.own.speeds          0.5310128  0.9118528

> plot(modswapB, results="hide", fig.show='hide')


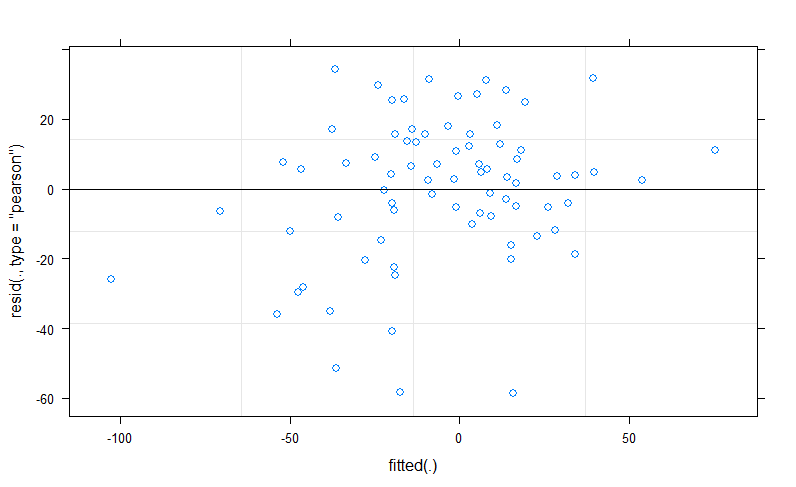


> qqmath(modswapB)


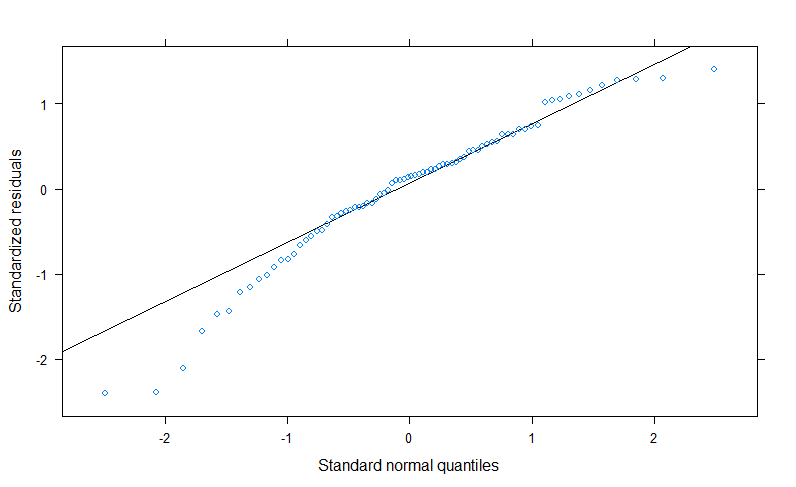


 > shapiro.test(resid(modswapB))
##
## Shapiro-Wilk normality test

## data:  resid(modswapB)
## W = 0.95397, p-value = 0.006723

> hist(resid(modswapB))


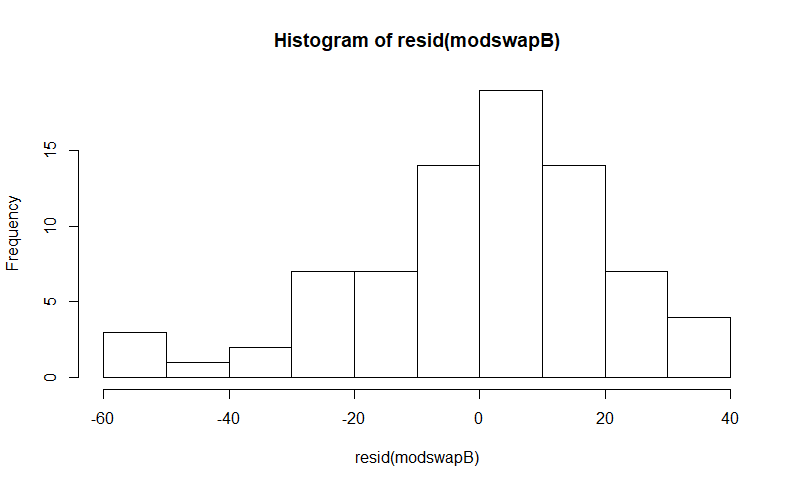


Has violated assumption of normality, will use transformation

| > Math.cbrt <- function(x) { +    sign(x) * abs(x)^(1/3)} #Create function for cube root transformation, this allows transformation of data to correct skew when including negative values, better option than applying a constant and then log transforming. |
| --- |
| > modswapC<-lmer(Math.cbrt(Difference.in.rivals.SF) ~ as.factor(YEAR) + SOCIAL.STATUS.SF + Difference.own.speeds  + (1\|pair) +(1\|WEEK)+(1\|Male.ID.sperm)+(1\|Rivals.ID),data=SEMINALFLUIDSWAP) > summary(modswapC) ## Linear mixed model fit by REML  ## t-tests use  Satterthwaite approximations to degrees of freedom  ## ['lmerMod'] ## Formula: Math.cbrt(Difference.in.rivals.SF) ~ as.factor(YEAR) +  ## SOCIAL.STATUS.SF +  Difference.own.speeds + (1 \| pair) + (1 \| WEEK) + ## (1 \| Male.ID.sperm) +  (1 \| Rivals.ID) ##  Data: SEMINALFLUIDSWAP  ## REML criterion at convergence: 352.3 ## ## Scaled residuals:  ##     Min       1Q   Median       3Q      Max  ## -2.31262 -0.62724  0.07258  0.69256  1.46382  ##  ## Random effects: ## Groups        Name        Variance Std.Dev. ## Rivals.ID     (Intercept) 0.7891   0.8883   ## Male.ID.sperm (Intercept) 0.8081   0.8989   ## pair          (Intercept) 0.0000   0.0000   ## WEEK          (Intercept) 0.0000   0.0000   ## Residual                  3.8187   1.9541   ## Number of obs: 78, groups:  Rivals.ID, 42; Male.ID.sperm, 42; pair, ## 39; WEEK, 5 ## ## Fixed effects: ##                      Estimate Std. Error  df t value Pr(>\|t\|)     ## (Intercept)           -0.643713  0.632289 34.1100  -1.018 0.316     ## as.factor(YEAR)2014    0.282424  0.723268 27.9300  0.390   0.699     ## as.factor(YEAR)2015    0.884680  0.776886 26.0100  1.139   0.265     ## SS.SFSubdominant       0.436635  0.594831 67.6900  0.734   0.465     ## Difference.own.speeds  0.050620  0.007802 69.1700   6.488  1.1e-08 *** ## --- ## Signif. codes:  0 ‘***’ 0.001 ‘**’ 0.01 ‘*’ 0.05 ‘.’ 0.1 ‘ ’ 1 ## ## Correlation of Fixed Effects: ##             (Intr) a.(YEAR)2014 a.(YEAR)2015 SS.SFS ## a.(YEAR)2014 -0.681                                  ## a.(YEAR)2015 -0.634  0.554                           ## SS.SFSbdmnn  -0.470  0.000        0.000              ## Dffrnc.wn.s   0.223  0.000        0.000       -0.475  > confint(modswapC, level=0.95, method="Wald",oldNames=F) #generate 95%CI using Wald method ##                                   2.5 %    97.5 % ## (Intercept)                  -1.88297629 0.5955510 ## as.factor(YEAR)2014          -1.13515432 1.7000027 ## as.factor(YEAR)2015          -0.63798882 2.4073494 ## SS.SFSubdominant             -0.72921148 1.6024819 ## Difference.own.speeds         0.03532906 0.0659114    > plot(modswapC, results="hide", fig.show='hide')  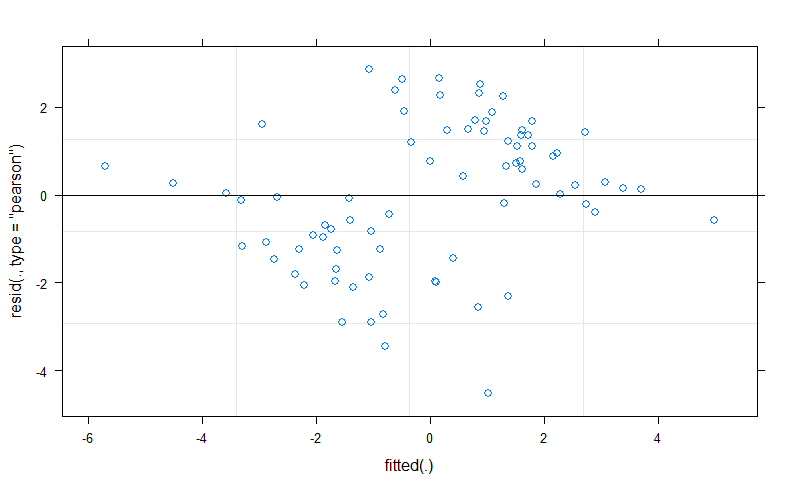 |
| > qqmath(modswapC)  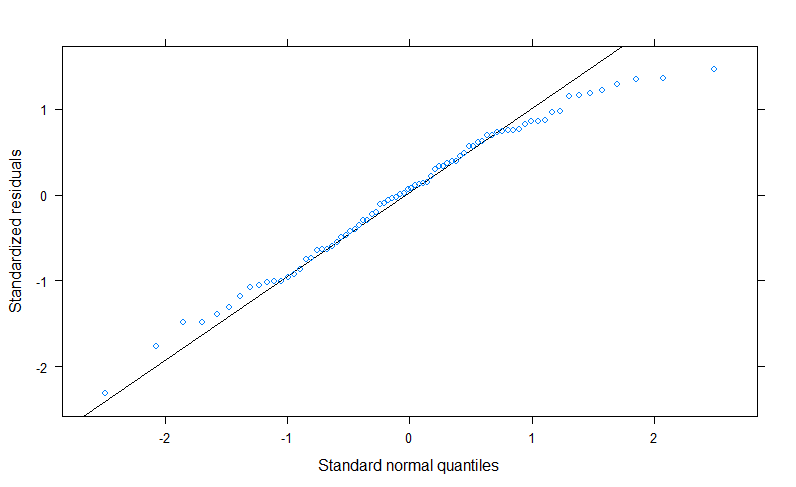   \| > shapiro.test(resid(modswapC)) ## ## Shapiro-Wilk normality test ## ## data:  resid(modswapC) ## W = 0.97744, p-value = 0.181  ## > hist(resid(modswapC)) \| \| --- \|   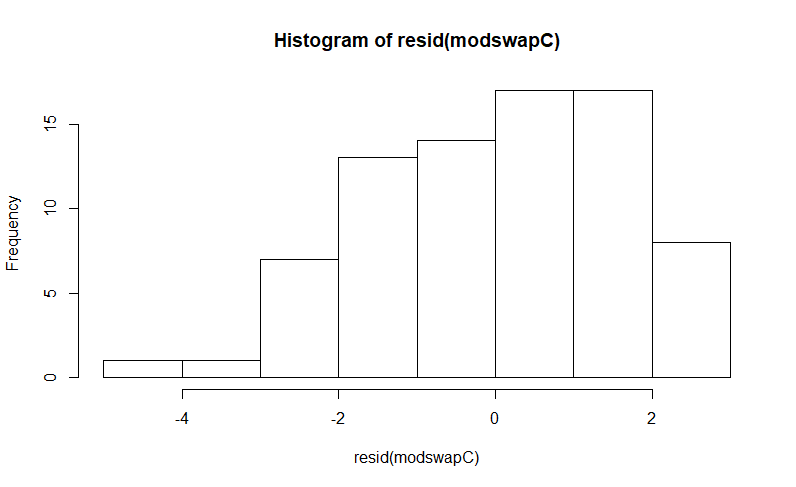 |

**Transformation appears to have pulled residuals towards normality, now** passes shapiro test.

**In-vitro fertilisation trials**

We used both Fishers exact and G tests to determine if the paternity shares observed across replicate fertility trials were repeatable.

Replicates<-read.table(file="FERT_TRIAL_REP_TEST.csv",header=T,row.names=NULL,sep=",")#load Data sheet

#Fisher exact and Likelihood ratio (G) tests

pval <- NULL

Gpval <- NULL

count1 <- 0

count2 <- 0

count3 <- 0

count4 <- 0

a <- 1

b <- 2

f1 <- NULL

mat1 <- NULL

for (i in 1:41) {

  mat1 <- Replicates[a:b,5:6]

  #print(mat1) #remove comment if you want to show each 2x2 matrix

  c1 <- fisher.test(mat1)

  c2 <- likelihood.test(mat1) #Pete Hurd's Likelihood Ratio (G-test) for contingency tables

  pval[i] <- c1$p.value #P from the Fisher tests

  Gpval[i] <- c2$p.value #P from the G-tests

  if(pval[i] <0.05) count1 <- count1+1

  if(pval[i] <0.0006) count2 <- count2+1

  if(Gpval[i] <0.05) count3 <- count3+1

  if(Gpval[i] <0.0006) count4 <- count4+1

  a=a+2

  b=b+2

}

> count1 #(pval[i] <0.05)
## [1] 2
> count2 #(pval[i] <0.0006)
## [1] 0
> count3 #(Gpval[i] <0.05)
## [1] 5
> count4 #(Gpval[i] <0.0006)
## [1] 1

#Adjusted p and G values using both holm and bonferroni methods

> p.adjust(pval, method="holm")
##[1] 1.0000000 1.0000000 1.0000000 1.0000000 1.0000000 1.0000000 ##1.0000000 1.0000000 1.0000000 1.0000000
##[11] 1.0000000 1.0000000 1.0000000 1.0000000 1.0000000 1.0000000 ##1.0000000 1.0000000 0.1598219 1.0000000
##[21] 1.0000000 1.0000000 1.0000000 1.0000000 1.0000000 1.0000000 ##1.0000000 1.0000000 1.0000000 1.0000000
##[31] 1.0000000 1.0000000 1.0000000 1.0000000 1.0000000 1.0000000 ##1.0000000 1.0000000 1.0000000 1.0000000
##[41] 1.0000000
> p.adjust(pval, method="bonferroni")
##[1] 1.0000000 1.0000000 1.0000000 1.0000000 1.0000000 1.0000000 ##1.0000000 1.0000000 1.0000000 1.0000000
##[11] 1.0000000 1.0000000 1.0000000 1.0000000 1.0000000 1.0000000 ##1.0000000 1.0000000 0.1598219 1.0000000
##[21] 1.0000000 1.0000000 1.0000000 1.0000000 1.0000000 1.0000000 ##1.0000000 1.0000000 1.0000000 1.0000000
##[31] 1.0000000 1.0000000 1.0000000 1.0000000 1.0000000 1.0000000 ##1.0000000 1.0000000 1.0000000 1.0000000
##[41] 1.0000000
> p.adjust(Gpval, method="holm")
##[1] 1.00000000 1.00000000 1.00000000 1.00000000 1.00000000 1.00000000 ##1.00000000 1.00000000 1.00000000
##[10] 1.00000000 1.00000000 1.00000000 1.00000000 1.00000000 1.00000000 ##1.00000000 1.00000000 1.00000000
##**[19] 0.01498229** 1.00000000 1.00000000 1.00000000 1.00000000 1.00000000 ##1.00000000 1.00000000 1.00000000
##[28] 1.00000000 1.00000000 1.00000000 1.00000000 1.00000000 0.24485998 ##1.00000000 1.00000000 1.00000000
##[37] 1.00000000 1.00000000 1.00000000 1.00000000 1.00000000
> p.adjust(Gpval, method="bonferroni")
##[1] 1.00000000 1.00000000 1.00000000 1.00000000 1.00000000 1.00000000 ##1.00000000 1.00000000 1.00000000
##[10] 1.00000000 1.00000000 1.00000000 1.00000000 1.00000000 1.00000000 ##1.00000000 1.00000000 1.00000000
##**[19] 0.01498229** 1.00000000 1.00000000 1.00000000 1.00000000 1.00000000 ##1.00000000 1.00000000 1.00000000
##[28] 1.00000000 1.00000000 1.00000000 1.00000000 1.00000000 0.25098148 ##1.00000000 1.00000000 1.00000000
##[37] 1.00000000 1.00000000 1.00000000 1.00000000 1.00000000

Replicates for trial 19 were removed from further analysis as paternity share differed significantly between replicates.

**Relative sperm velocity as a predictor of fertilisation success (in both seminal fluid treatments)**

In-vitro fertilisation trials using unmanipulated milt

>TRIALSM2<-read.table(file="FERT_REL_VAP_MILT.csv",header=T,row.names=NULL,sep=",")#load Data sheet

> TRIALSNEW<-TRIALSM2[c(-38,-39),] #remove trial 19 from the data that failed the replicability tests (see above)

> #does relative sperm velocity (measured in OF) predict fert success?

## Linear mixed model fit by REML

## t-tests use Satterthwaite approximations to degrees of freedom
## ['lmerMod']

## Formula: DiffFERTmaleA ~ YEAR + DiffVAPof + (1 | WEEK) + (1 | maleA) +

## (1 | maleB) + (1 | Female) + (1 | TRYAD)

## Data: TRIALSNEW

##

##REML criterion at convergence: 240.5

##

## Scaled residuals:

## Min 1Q Median 3Q Max

## -1.80443 -0.63631 0.04371 0.45178 1.74778

##

## Random effects:

## Groups Name Variance Std.Dev.

## TRYAD (Intercept) 1.054e+01 3.247e+00

## Female (Intercept) 1.836e-07 4.285e-04

## maleA (Intercept) 2.077e+01 4.558e+00

## maleB (Intercept) 3.743e-07 6.118e-04

## WEEK (Intercept) 2.130e-09 4.615e-05

## Residual 1.044e+01 3.231e+00

## Number of obs: 40, groups: TRYAD, 21; Female, 17; maleA, 17; maleB, 15## ; WEEK, 4

## Fixed effects:

## Estimate Std. Error df t value Pr(>|t|)

## (Intercept) -1.488e+03 5.774e+03 1.998e+01 -0.258 0.79926

## YEAR 7.419e-01 2.866e+00 1.998e+01 0.259 0.79842

## diffVAPof 1.443e-01 4.313e-02 2.542e+01 3.345 0.00256 **

## ---

## Signif. codes: 0 ‘***’ 0.001 ‘**’ 0.01 ‘*’ 0.05 ‘.’ 0.1 ‘ ’ 1

## Correlation of Fixed Effects:

## (Intr) YEAR

## YEAR -1.000

## diffVAPof -0.057 0.057

> confint(modA.2, level=0.95, method="Wald",oldNames=F) #generate 95%CI using Wald method
##                           2.5 %     97.5 %
## (Intercept) -1.280511e+04 9828.925960

## YEAR -4.875879e+00 6.359599

## diffVAPof 5.975185e-02 0.228811

> qqmath(modA.2)
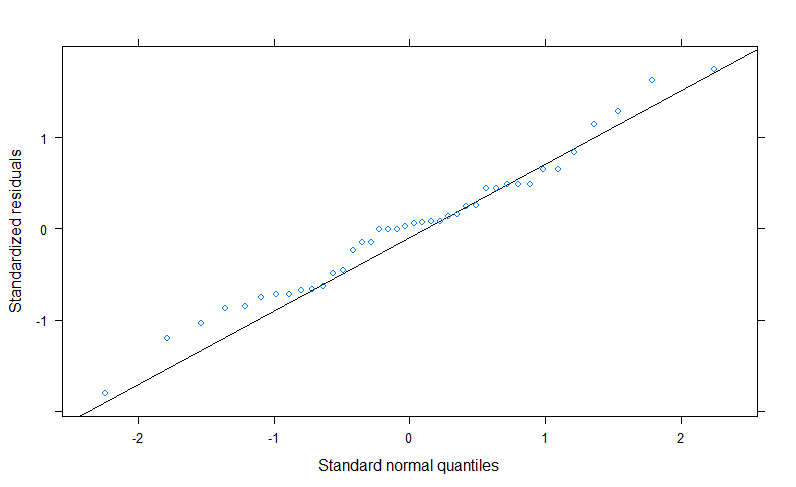


 > shapiro.test(resid(modA.2))

## Shapiro-Wilk normality test
##
## data: resid(modA.2)

## W = 0.98044, p-value = 0.7059

> plot(modA.2, results="hide", fig.show='hide')


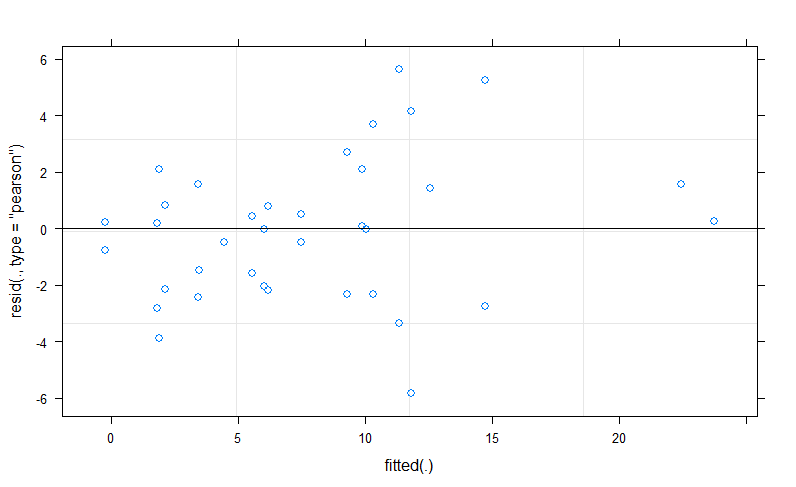


In-vitro fertilisation trials using manipulated milt (seminal fluid and sperm swapped between focal and rival male in each trial).

>TRIALSSWAP<-read.table(file="FERT_REL_VAP_SWAP.csv",header=T,row.names=NULL,sep=",")
#load Data sheet

> #does relative sperm velocity (measured in OF) predict fert success?

## Linear mixed model fit by REML

## t-tests use Satterthwaite approximations to degrees of freedom
## ['lmerMod']

## Formula: DiffFERTmaleA ~ YEAR + DiffVAPof + (1 | WEEK) + (1 | maleA) +

## (1 | maleB) + (1 | Female) + (1 | TRYAD)

## Data: TRIALSSWAP

##

## REML criterion at convergence: 265.8

##

## Scaled residuals:

## Min 1Q Median 3Q Max

## -2.0423 -0.7212 0.2128 0.5847 1.8003

##

## Random effects:

## Groups Name Variance Std.Dev.

## TRYAD (Intercept) 1.002e-07 0.0003165

## Female (Intercept) 2.889e+01 5.3752530

## maleB (Intercept) 2.991e-06 0.0017296

## maleA (Intercept) 0.000e+00 0.0000000

## WEEK (Intercept) 0.000e+00 0.0000000

## Residual 2.108e+01 4.5908229

## Number of obs: 42, groups: TRYAD, 21; Female, 16; maleB, 16; maleA,
## 16; WEEK, 4

##

## Fixed effects:

## Estimate Std. Error df t value Pr(>|t|)

## (Intercept) 3722.73905 6293.48426 19.22000 0.592 0.56106

## YEAR -1.84273 3.12406 19.22000 -0.590 0.56216

## diffVAPof 0.13489 0.04216 36.55000 3.200 0.00284 **

## ---

## Signif. codes: 0 ‘***’ 0.001 ‘**’ 0.01 ‘*’ 0.05 ‘.’ 0.1 ‘ ’ 1

##

## Correlation of Fixed Effects:

## (Intr) YEAR

## YEAR -1.000

## diffVAPof 0.157 -0.157

> confint(modB.2, level=0.95, method="Wald",oldNames=F) #generate 95%CI using Wald method
##                          2.5 %      97.5 %
## (Intercept) -8.612263e+03 1.605774e+04

## YEAR -7.965773e+00 4.280319e+00
## diffVAPof 5.226232e-02 2.175134e-01

> qqmath(modB.2)

  
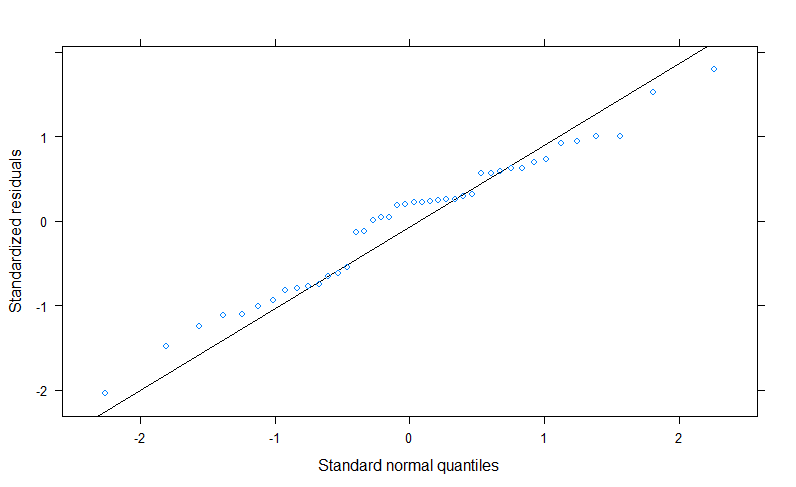


> shapiro.test(resid(modB.2))

## Shapiro-Wilk normality test
##
## data: resid(modB.2)

## W = 0.97372, p-value = 0.4366

> plot(modB.2, results="hide", fig.show='hide')


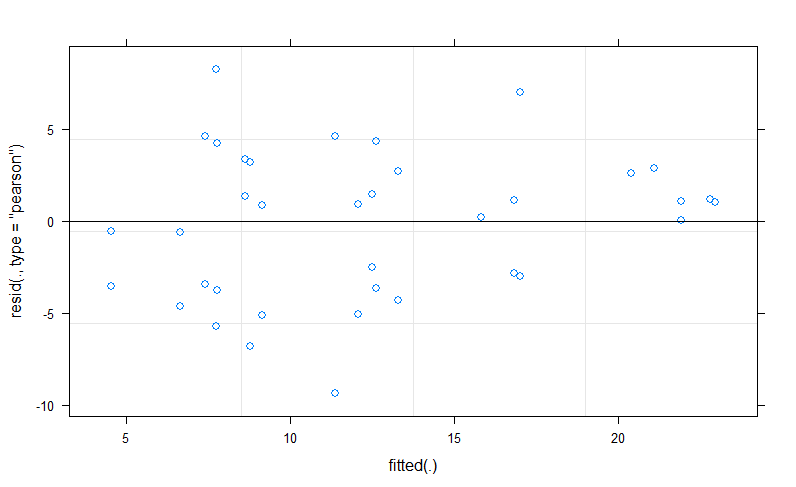


**Comparing the proportion of offspring sired by males of different social status (in both seminal fluid treatments)**

unmanipulated milt trials:

TRIALS<-
read.table(file="FERT_SS_MILT.csv",header=T,row.names=NULL,sep=",") #load Data sheet

TNEW <-TRIALS[c(-75,-76,-77,-78),] #remove trial 20 from the data that failed the replicability tests (see above)

> modX<-glmer(PFERT~SS+(1|MaleID)+(1|Female)+(1|TRYAD)+(1|WEEK),
 family="binomial", weights=TOTAL ,data=TNEW)
> summary(modX)
## Generalized linear mixed model fit by maximum likelihood (Laplace
## Approximation) ['glmerMod']
## Family: binomial  ( logit )
## Formula: PFERT ~ SS + (1 | MaleID) + (1 | Female) + (1 | TRYAD) +
## (1 | WEEK)
## Data: TNEW
## Weights: TOTAL
##
##    AIC      BIC   logLik deviance df.resid
##   467.8    482.1   -227.9    455.8       74
##
## Scaled residuals:
##    Min       1Q   Median       3Q      Max
## -2.06803 -0.43276  0.00992  0.45587  1.69527
##
## Random effects:
## Groups Name        Variance  Std.Dev.
## MaleID (Intercept) 2.156e+00 1.468e+00
## TRYAD  (Intercept) 5.400e-01 7.348e-01
## Female (Intercept) 1.311e-09 3.621e-05
## WEEK   (Intercept) 4.502e-09 6.710e-05
## Number of obs: 80, groups:  MaleID, 24; TRYAD, 21; Female, 17; WEEK, 4
##
## Fixed effects:
##           Estimate Std. Error z value Pr(>|z|)    
## (Intercept)  -0.3789     0.3655  -1.037      0.3    
## SSS           1.1052     0.2400   4.604 4.14e-06 ***
## ---
## Signif. codes:  0 ‘***’ 0.001 ‘**’ 0.01 ‘*’ 0.05 ‘.’ 0.1 ‘ ’ 1
##
## Correlation of Fixed Effects:
##   (Intr)
## SSS -0.320
> confint(modX, level=0.95, method="Wald",oldNames=F)
##                          2.5 %    97.5 %
## (Intercept)           -1.0952987 0.3375164
## SSS                    0.6347259 1.5756610

> overdisp.glmer(modX)# check for over/under-dispersion
## Residual deviance: 63.209 on 74 degrees of freedom (ratio: 0.854)

Seminal fluid swapped trials:

>TRIALS2<-
read.table(file="FERT_SS_SWAP.csv",header=T,row.names=NULL,sep=",")
#load Data sheet
> #Swapped seminal fluid trials, social status of seminal fluid in
 which sperm were incubated as fixed predictor
> modX2<-glmer(PFERT~SS.SF+(1|maleID)+(1|Female)+(1|TRYAD)+(1|WEEK),
family="binomial", weights=TOTAL ,data=TRIALS2)
> summary(modX2)
## Generalized linear mixed model fit by maximum likelihood (Laplace
## Approximation) ['glmerMod']
## Family: binomial  ( logit )
## Formula: PFERT ~ SS.SF + (1 | maleID) + (1 | Female) + (1 | TRYAD) +
## (1 | WEEK)
##  Data: TRIALS2
## Weights: TOTAL
##
##    AIC      BIC   logLik deviance df.resid
##  537.1    551.7   -262.5    525.1       78
##
## Scaled residuals:
##     Min       1Q   Median       3Q      Max
## -2.41280 -0.55202 -0.02723  0.58103  2.36920
##
## Random effects:
## Groups Name        Variance Std.Dev.
## maleID (Intercept) 25.29    5.029   
## TRYAD  (Intercept) 13.87    3.725   
## Female (Intercept)  0.00    0.000   
## WEEK   (Intercept)  0.00    0.000   
## Number of obs: 84, groups:  maleID, 24; TRYAD, 21; Female, 16; WEEK, 4
##
## Fixed effects:
##           Estimate Std. Error z value Pr(>|z|)    
## (Intercept)   -3.241      1.381  -2.347   0.0189 *  
## SS.SFS         6.225      0.766   8.126 4.44e-16 ***
## ---
## Signif. codes:  0 ‘***’ 0.001 ‘**’ 0.01 ‘*’ 0.05 ‘.’ 0.1 ‘ ’ 1
##
## Correlation of Fixed Effects:
##      (Intr)
## SS.SFS -0.283
> confint(modX2, level=0.95, method="Wald",oldNames=F)
##                         2.5 %     97.5 %
## (Intercept)           -5.947186 -0.5339593
## SS.SFS                 4.723309  7.7261285
> overdisp.glmer(modX2)# check for over/under-dispersion
## Residual deviance: 86.337 on 78 degrees of freedom (ratio: 1.107)

**Testing relationship between the change in number of eggs fertilised and the change in relative sperm velocity across seminal fluid treatments within the same male-male-female combinations.**

>TREATEFF<-read.table(file="DIFF_ACROSS_TREATMENTS.csv",header=T,row.names=NULL,sep=",")#load Data sheet

> TRIALSNEW2<-TREATEFF[c(-19),] #remove trial 20 from the data that failed the replicability tests (see above)

> modC<-lmer(DIFFPFERT ~ DIFFVAPof + YEAR+ (1|maleA)+(1|maleB)+(1|WEEK),data=TRIALSNEW2)

> summary(modC)

## Linear mixed model fit by REML t-tests use Satterthwaite

## approximations to degrees of freedom [lmerMod]

## Formula:

## DIFFPFERT ~ DIFFVAPof + YEAR + (1 | maleA) + (1 | maleB) +
## (1 | WEEK)

## Data: TRIALSNEW2

##

## REML criterion at convergence: 18.3

##

## Scaled residuals:

##   Min      1Q   Median    3Q     Max

## -1.7865 -0.3430  0.1815  0.5275  0.9868

##

## Random effects:

## Groups   Name        Variance Std.Dev.

## maleA    (Intercept) 0.022522 0.15007

## maleB    (Intercept) 0.004506 0.06713

## WEEK     (Intercept) 0.000000 0.00000

## Residual             0.043366 0.20824

## Number of obs: 20, groups:  maleA, 17; maleB, 15; WEEK, 4

##

## Fixed effects:

##              Estimate Std. Error     df t value Pr(>|t|)

## (Intercept) -56.398420 252.018591  14.297000  -0.224 0.826090

## DIFFVAPof     0.005577   0.001034  12.997000   5.395 0.000122 ***

## YEAR          0.028045   0.125102  14.297000   0.224 0.825788

## ---

## Signif. codes:  0 ‘***’ 0.001 ‘**’ 0.01 ‘*’ 0.05 ‘.’ 0.1 ‘ ’ 1

##

## Correlation of Fixed Effects:

##          (Intr) DIFFVA

## DIFFVAPof -0.045

## YEAR      -1.000  0.045

> confint(modC, level=0.95, method="Wald",oldNames=F) #generate 95%CI using Wald method

##                            2.5 %        97.5 %

## (Intercept)          -5.503458e+02 4.375489e+02

## DIFFVAPof             3.550875e-03 7.603404e-03

## YEAR                 -2.171491e-01 2.732401e-01

> qqmath(modC)


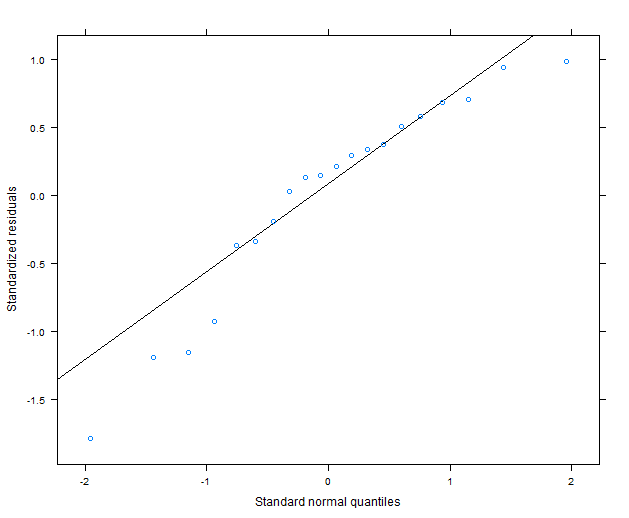


> shapiro.test(resid(modC))

## Shapiro-Wilk normality test

##

## data:  resid(modC)

## W = 0.91891, p-value = 0.09442

> plot(modC, results="hide", fig.show='hide')


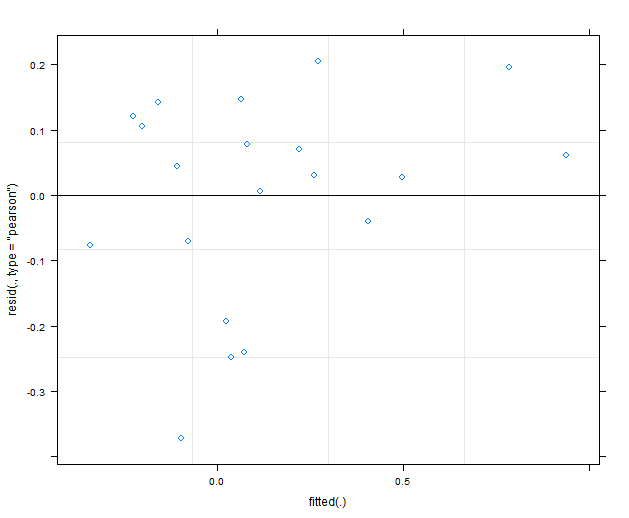


**References**

[1] R Core Team (2016) R: A language and environment for statistical computing. R Foundation for Statistical Computing, Vienna, Austria.

URL: https://www.R-project.org/

[2] Wickham H. (2009) ggplot2: Elegant Graphics for Data Analysis. Springer-Verlag New York. doi:10.1007/978-0-387-98141-3

[3] Sarkar D. (2008) Lattice: Multivariate Data Visualization with R. Springer, New York. ISBN 978-0-387-75968-5

[4] Bates D., Maechler M., Bolker B. & Walker S. (2015). Fitting Linear Mixed-Effects Models Using lme4. *Journal of Statistical Software*, 67(1), 1-48. doi:10.18637/jss.v067.i01.

[5] Pinheiro J., Bates D., DebRoy S., Sarkar D. & R Core Team (2015). nlme: Linear and Nonlinear Mixed Effects Models. R package v 3.1-121.

URL: http://CRAN.R-project.org/package=nlme

[6] Kuznetsova A., Brockhoff P.B. & Christensen R. (2016). lmerTest: Tests in Linear Mixed Effects Models. R package v 2.0-30.

URL: http://CRAN.R-project.org/package=lmerTest

[7] Hervé M. (2016). RVAideMemoire: Diverse Basic Statistical and Graphical Functions. R package v 0.9-55.

URL: http://CRAN.R-project.org/package=RVAideMemoire

[8] Tremblay A. & Ransijn J. (2015). LMERConvenienceFunctions: Model Selection and Post-hoc Analysis for (G)LMER Models. R package v 2.10.

URL: http://CRAN.R-project.org/package=LMERConvenienceFunctions

[9] Fellows I. (2012). Deducer: A Data Analysis GUI for R. Journal of Statistical Software, 49(8), p. 1-15. doi:10.18637/jss.v049.i08

URL http://www.jstatsoft.org/v49/i08/.

[10] Bolker BM. (2015) Linear and generalized linear mixed models. In: Fox GA, Negrete-Yankelevich S, Sosa VJ, editors. Ecological Statistics: Contemporary Theory and Application. 1st ed. Oxford University Press. p. 309–333.
